# SQSTM1/p62 mediates crosstalk between autophagy and the UPS in DNA repair

**DOI:** 10.1080/15548627.2016.1210368

**Published:** 2016-07-08

**Authors:** Graeme Hewitt, Bernadette Carroll, Rezazadeh Sarallah, Clara Correia-Melo, Mikołaj Ogrodnik, Glyn Nelson, Elsje G. Otten, Diego Manni, Robin Antrobus, Brian A. Morgan, Thomas von Zglinicki, Diana Jurk, Andrei Seluanov, Vera Gorbunova, Terje Johansen, João F. Passos, Viktor I. Korolchuk

**Affiliations:** a Institute for Cell and Molecular Biosciences, Newcastle University, Newcastle upon Tyne, UK; b Cambridge Institute for Medical Research, Cambridge University, Cambridge, UK; c Department of Biology, University of Rochester, Rochester, NY USA; d Molecular Cancer Research Group, Department of Medical Biology, University of Tromsø – The Arctic University of Norway, Tromsø, Norway

**Keywords:** aging, autophagy, DNA damage, homologous recombination, nonhomologous end joining, SQSTM1

## Abstract

SQSTM1/p62 (sequestosome 1) selectively targets polyubiquitinated proteins for degradation via macroautophagy and the proteasome. Additionally, SQSTM1 shuttles between the cytoplasmic and nuclear compartments, although its role in the nucleus is relatively unknown. Here, we report that SQSTM1 dynamically associates with DNA damage foci (DDF) and regulates DNA repair. Upon induction of DNA damage SQSTM1 interacts with FLNA (filamin A), which has previously been shown to recruit DNA repair protein RAD51 (RAD51 recombinase) to double-strand breaks and facilitate homologous recombination (HR). SQSTM1 promotes proteasomal degradation of FLNA and RAD51 within the nucleus, resulting in reduced levels of nuclear RAD51 and slower DNA repair. SQSTM1 regulates the ratio between HR and nonhomologous end joining (NHEJ) by promoting the latter at the expense of the former. This SQSTM1-dependent mechanism mediates the effect of macroautophagy on DNA repair. Moreover, nuclear localization of SQSTM1 and its association with DDF increase with aging and are prevented by life-span-extending dietary restriction, suggesting that an imbalance in the mechanism identified here may contribute to aging and age-related diseases.

## Introduction

The DNA damage response (DDR) is essential for the maintenance of genome stability and its impairment is implicated in human diseases and aging.^1,2^ The role of the ubiquitin-proteasome system (UPS) in the control of DDR pathways is well established.^3^ Proteasomal degradation plays the key role in the turnover of the components of the DNA repair machinery during genome maintenance and promotes efficient resolution of DNA damage foci (DDF), thus allowing cell cycle progression. Recently, lysosome-dependent degradation pathways, including macroautophagy (hereinafter autophagy) and chaperone-mediated autophagy (CMA) have also been demonstrated to affect the DDR in eukaryotic cells. Specifically, suppression of autophagy results in an aberrant DDR with delayed kinetics of DNA repair and increased dependence on NHEJ at the expense of HR.^4-6^

Autophagy is thought to take place exclusively in the cytoplasm while DDR is a primarily nuclear process. Several mechanisms have been proposed as a link between these 2 pathways. In some specific physiological contexts, such as deep cellular senescence, transport of DDF from the nucleus into the cytoplasm followed by engulfment by autophagic membranes has been observed.^7,8^ Alternatively, the positive effect of autophagy on DNA repair may be achieved by the transport of specific nuclear proteins into the cytoplasm where they are recruited for autophagic degradation. Specifically, it has been suggested that acetylation-dependent autophagic turnover of the recombination protein Sae2 promotes DNA repair in yeast^6^ although this mechanism has recently been challenged in mammalian cells; turnover of CHEK1 (checkpoint kinase 1) via either autophagy or CMA has been proposed to mediate the positive role of these degradative pathways in DDR.^4,9^ However, while the latter mechanism helps to explain how autophagy could affect signaling downstream of DDF the mechanisms allowing autophagy to promote DNA repair remain poorly understood. Different mechanisms of double-strand DNA damage repair are controlled by dedicated molecular machinery and RAD51 and TP53BP1 (tumor protein p53 binding protein 1) have been shown to promote HR and NHEJ, respectively. A differential effect of autophagy on HR and NHEJ raises a possibility that autophagy has a direct or an indirect effect on the levels or activity of these proteins.^10^

In eukaryotic cells activity of autophagy and UPS are tightly regulated and coordinated. Thus, impairment of proteasomal degradation can be compensated by upregulation of autophagy, whereas inhibition of autophagy has been proposed to suppress proteasomal degradation.^11^ We and others previously demonstrated that this crosstalk can, at least in part, be mediated by a prototypical autophagic substrate and receptor protein, SQSTM1. SQSTM1 contains a ubiquitin-associated (UBA) domain which recognizes substrates targeted for degradation via both autophagy and proteasomal pathways.^12^ We have also recently demonstrated that SQSTM1 rapidly shuttles between the cytoplasmic and nuclear compartments. Although it has been shown that SQSTM1 is involved in proteasomal degradation of aggregate-prone nuclear proteins, the precise functional role(s) of SQSTM1 in the nucleus remains to be elucidated.^13^

Here we show that SQSTM1 acts as a mechanistic link between autophagy, the UPS and DNA repair. Nuclear SQSTM1 facilitates proteasomal degradation of DNA repair machinery components, including FLNA and RAD51, and knockout of SQSTM1 increases the rate of DNA repair and specifically promotes HR. The positive effect of autophagy on DNA repair is mediated by SQSTM1 and this mechanism may help to explain the reduced efficiency of DNA repair and an increased reliance on NHEJ in aged cells and tissues, which are characterized by an impairment in the autophagy pathway. Additionally, this mechanism helps to link accumulation of SQSTM1, identified in many human age-related diseases including neurodegenerative disorders and cancers, with reduced DNA repair and genome instability.^1^

## Results

### Nuclear SQSTM1 colocalizes with DDF

**Figure 1.**
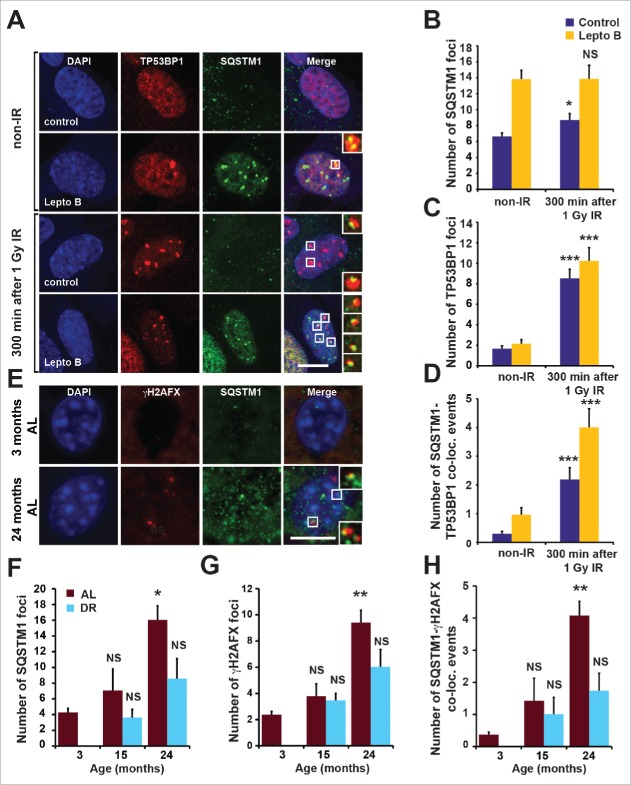
Nuclear SQSTM1 colocalizes with DDF. (A-D) The colocalization of SQSTM1 and TP53BP1 was analyzed in human fibroblasts (MRC5) exposed to irradiation (IR) for 0 and 5 h in the absence or presence of leptomycin B (Lepto B) as indicated. Representative images are shown in (A) and the mean number of SQSTM1 (B), TP53BP1 (C) and SQSTM1-TP53BP1 colocalization (D) foci were quantified. (E) Representative images of hepatocytes from young (3-m–old) and old (24-mo-old) male C57BL/6 wild-type mice maintained under *ad libitum* (AL) diet. Sections were immunostained with antibodies against SQSTM1 and γH2AFX. The mean number of SQSTM1 (F), γH2AFX (G) and SQSTM1-γH2AFX colocalization (D) foci were quantified in hepatocyte sections from mice maintained under AL or dietary-restricted (DR) diets. Scale bars: 10 µm; n = 3; error bars represent SEM; *, p < 0.05; **, p < 0.01; ***, p < 0.001.

Although the signaling adaptor and autophagic receptor protein SQSTM1 is known to shuttle between nuclear and cytoplasmic compartments,^13^ its nuclear role is unclear. Interestingly, we found that SQSTM1 associates with markers of DNA damage. Thus, a fraction of TP53BP1- or phosphorylated H2AFX (H2A histone family member X; γH2AFX)-positive DNA damage foci (DDF) induced by 1 Gy X-ray irradiation (IR) in cultured human fibroblasts contained SQSTM1 (Fig. 1A,B and Fig. S1A). Moreover, this colocalization was significantly more pronounced upon inhibition of nuclear export of SQSTM1 by leptomycin B (Fig. 1A,B and Fig. S1A), suggesting a transient association of SQSTM1 with DDF. The number of nuclear SQSTM1 foci was only mildly increased by IR (an effect not seen in the presence of leptomycin B) and did not respond to the suppression of DDR signaling pathways mediated by ATM (serine/threonine kinase) and ATR (ATR serine/threonine kinase) (Fig. S1B-G).^13^ This constitutive nucleocytoplasmic SQSTM1 shuttling is in agreement with our previous finding that SQSTM1 associates with promyelocytic leukemia bodies.^13,14^

Additionally, DDF in mouse liver and intestinal tissues were found to partially colocalize with SQSTM1 (Fig. S1H). Nuclear SQSTM1, as well as its colocalization with γH2AX-positive DDF, was increasingly evident with aging (Fig. 1E–H and Fig. S1I,J). Moreover, dietary restriction (DR), which promotes autophagy and consequently SQSTM1 degradation, reduced the frequency of nuclear SQSTM1- and γH2AX-positive DDF compared to an *ad libitum* (AL) feeding regimen (Fig. 1E–H and Fig. S1I,J).^13^ Together these observations suggest that SQSTM1 is dynamically recruited to DDF and may play a role in DNA repair. Furthermore, clearance of nuclear SQSTM1 foci following DR correlates with the reduction of DDF suggesting a role for SQSTM1 in mediating the effect of autophagy on DNA repair.

### SQSTM1 suppresses the rate of DNA repair

**Figure 2.**
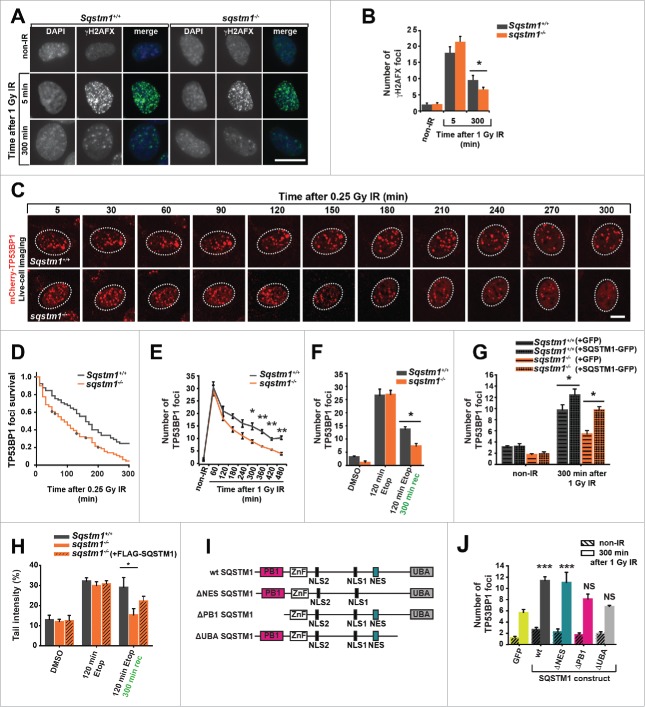
SQSTM1 suppresses resolution of TP53BP1-positive DDF. Representative images (A) and quantification (B) of the mean number of γH2AFX foci in *sqstm1*^*−/−*^ and *Sqstm1*^*+/+*^ MEFs in non-IR and 5 and 300 min following 1 Gy X-ray irradiation. (C-D) *sqstm1*^*−/−*^ and *Sqstm1*^*+/+*^ MEFs stably expressing mCherry-TP53BP1 were exposed to 0.25 Gy X-ray irradiation and TP53BP1 foci kinetics were monitored by live cell imaging for 300 min. Representative images are shown in (C), the nucleus is marked by a dotted white border. (D) Kaplan-Meier plot showing the survival of individual TP53BP1 foci in *sqstm1*^*−/−*^ and *Sqstm1*^*+/+*^ MEFs following irradiation. Note that 0.25 Gy was used to induce a low frequency of DDF and facilitate accurate tracking of foci in vivo. Gehan-Breslow test p<0.01. (E) Quantification of the mean number of endogenous TP53BP1 foci in *sqstm1*^*−/−*^ and *Sqstm1*^*+/+*^ MEFs 0-480 min following 1 Gy X-ray irradiation. (F) Quantification of the mean number of endogenous TP53BP1 foci in *sqstm1*^*−/−*^ and *Sqstm1*^*+/+*^ MEFs following the induction of DNA damage with etoposide (Etop) for 120 min followed either with or without a 300-min recovery period (in the absence of etoposide). (G) Quantification of the mean number of TP53BP1 foci 300 min after irradiation of *sqstm1*^*−/−*^ and *Sqstm1*^*+/+*^ MEFs transfected overnight with control (GFP) or GFP-SQSTM1 plasmids. (H) *Sqstm1*^*+/+*^ MEFs, *sqstm1*^*−/−*^ MEFs, and *sqstm1*^*−/−*^ MEFs overexpressing FLAG-SQSTM1 were treated with etoposide for 120 min either with or without a 300-min recovery period where indicated. Samples were subjected to neutral comet and the percent tail intensity quantified. (I) Schematic representation of the domain structure of SQSTM1 constructs. Key structural domains are marked: UBA, ubiquitin-associated domain; PB1, Phox and Bem1p domain; ZnF, ZZ type zinc finger domain; NES, nuclear export signal; NLS1/2, nuclear localization signal. (J) Quantification of the mean number of TP53BP1 foci in *sqstm1*^*−/−*^ MEFs overexpressing the indicated GFP-tagged SQSTM1 mutants 300 min after irradiation. Scale bar: 10 µm. n = 3; error bars represent SEM; NS, not significant; *, p < 0.05; **, p < 0.01; ***, p < 0.001.

In order to investigate the role of SQSTM1 in DNA repair we subjected *Sqstm1*^*+/+*^ and *sqstm1*^*−/−*^ mouse embryonic fibroblasts (MEFs) to X-ray irradiation and quantified the number of γH2AFX-positive DDF immediately (5 min) and 300 min after the induction of DNA damage repair.^15^ The absence of SQSTM1 did not affect the number of DDF immediately after IR suggesting that SQSTM1 is not involved in the initial stages of the DDR. However, the number of foci following repair was reduced in cells lacking SQSTM1 (Fig. 2A,B). In order to track foci dynamics using live-cell imaging we utilized *Sqstm1*^*+/+*^ and *sqstm1*^*−/−*^ MEFs stably expressing the DDF marker mCherry-TP53BP1. Cells lacking SQSTM1 showed a significantly shorter TP53BP1 foci life span (Fig. 2C,D). Similarly, *sqstm1*^*−/−*^ MEFs subjected to X-ray irradiation and fixed at different time points showed an increased TP53BP1 foci resolution compared to wild-type cells (Fig. 2E and Fig. S2A). As with γH2AX-positive DDF, absence of SQSTM1 did not affect the frequency of mCherry-TP53BP1 foci formation after X-ray irradiation (Fig. 2A–E and Fig. S2A). We also investigated the effect of SQSTM1 on DNA repair in response to another genotoxic agent, the topoisomerase II inhibitor etoposide.^16^ Similar to IR treatment, the presence or absence of SQSTM1 did not affect the initial stages of the response to etoposide, but the presence of SQSTM1 was found to delay the clearance of TP53BP1-positive DDF (Fig. 2F and Fig. S2B). Confirming a role for SQSTM1 in this process, transient expression of GFP-SQSTM1 rescued the number of DDF in *sqstm1*^*−/−*^ MEFs, whereas overexpressed GFP-SQSTM1 increased the frequency of DDF further (Fig. 2G).

To investigate an effect of SQSTM1 on DNA repair directly we used a neutral comet assay, which measures the frequency of DNA breaks.^17^ Similar to DDF kinetics, the loss of SQSTM1 enhanced the rate of DNA repair following etoposide treatment, whereas stable expression of FLAG-tagged SQSTM1 could partially suppress the faster rate of DNA repair observed in *sqstm1*^*−/−*^ MEFs (Fig. 2H and Fig. S2C,D). The partial effect of stably expressing FLAG-tagged SQSTM1 is likely due to lower expression levels of the transgenic FLAG-SQSTM1 construct compared to the endogenous protein (Fig. S2C). To investigate the effect of SQSTM1 on processes downstream of DNA damage, we knocked down *SQSTM1* in primary human fibroblasts and measured the rate at which cells escape cell cycle arrest following IR using a 5-ethynyl-2′-deoxyuridine (EdU) incorporation assay. In agreement with faster DNA damage resolution in cells lacking SQSTM1, knockdown of *SQSTM1* also facilitated the exit from cell cycle arrest (Fig. S2E-G). Together, these data indicate that nuclear SQSTM1 reduces the rate of DNA repair as measured by the frequency of γH2AX- and TP53BP1-positive foci and by comet assays and acts as a checkpoint for cell cycle progression following DNA damage.

To identify the domain(s) of SQSTM1 important for its function in DNA repair we compared the ability of full-length GFP-SQSTM1 to inhibit the disappearance of DDF with SQSTM1 mutants lacking either the Phox and Bem1 (PB1) domain responsible for protein oligomerization (ΔPB1), the UBA domain involved in binding to ubiquitinated substrates (ΔUBA), or the nuclear export signal (ΔNES) (Fig. 2I). Interestingly, while both the PB1 and the UBA domain appeared to affect the frequency of DDF, the NES was dispensable suggesting that SQSTM1 does not need to be shuttled from the nucleus into the cytoplasm to have an effect on this process (Fig. 2J).

### Regulation of DDF resolution kinetics by autophagy is dependent on SQSTM1

**Figure 3.**
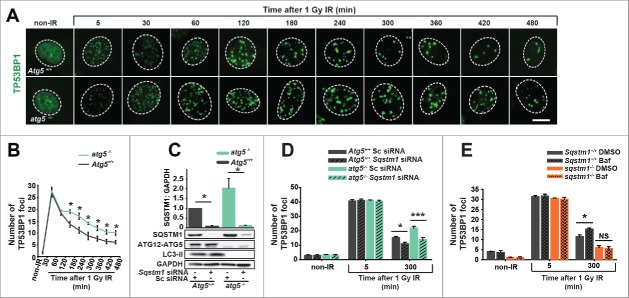
SQSTM1 mediates the effect of autophagy on DNA repair. Representative images (A) and quantification (B) of the mean number of TP53BP1 foci in *Atg5*^*+/+*^ and *atg5*^*−/−*^ MEFs 0-480 min after irradiation. (C) Blot showing scrambled control (Sc) and *Sqstm1* siRNA in *Atg5*^*+/+*^ and *atg5*^*−/−*^ MEFs. (D) Quantification of the mean number of endogenous TP53BP1 foci in *Atg5*^*+/+*^ and *atg5*^*−/−*^ MEFs treated with control or *Sqstm1* siRNA 5 and 300 min after irradiation. (E) Quantification of the mean number of endogenous TP53BP1 foci in *Sqstm1*^*+/+*^ and *sqstm1*^*−/−*^ MEFs treated with or without bafilomycin A_1_ (Baf) 5 and 300 min after irradiation. Scale bar: 10 µm; n = 3; error bars represent SEM; NS, not significant; *, p < 0.05; **, p < 0.01; ***, p < 0.001.

Inhibition of autophagy by knockout of genes encoding ATG7 (autophagy-related 7) and RB1CC1/FIP200 (RB1 inducible coiled-coil 1) has previously been demonstrated to suppress DNA repair.^4-6^ We validated these findings using knockout of another essential autophagy gene, *Atg5*. Indeed, resolution of DDF following IR was found to be delayed in cells with constitutive or inducible knockout of *Atg5* compared to cells with functional autophagy (Fig. 3A,B and Fig. S3A,B). Furthermore, *Atg5* knockout prevented efficient clearance of DDF induced by etoposide treatment (Fig. S3C,D). The initial formation of DDF was indistinguishable between autophagy-competent and -defective cells (Fig. 3A,B and Fig. S3A-D) suggesting that the amount of DNA damage caused by either IR or etoposide is not different between cell lines. Small, statistically nonsignificant upregulation of autophagy flux was observed following IR (not shown).

Since SQSTM1 is one of the most prominent autophagy substrates and can regulate the rate of DNA repair, we next investigated if the effect of autophagy on DNA repair is mediated by this protein.^18^ Indeed, knockdown of *Sqstm1* efficiently increased the clearance of DDF in both wild-type and *atg5*^*−/−*^ cells and, furthermore, loss of autophagy did not have any significant effect on DDF in the absence of SQSTM1 (Fig. 3C,D and Fig. S3E). Finally, treatment of cells with the lysosomal inhibitor bafilomycin A_1_ (Baf) was used as an alternative method of autophagy inhibition, as indicated by an increase in MAP1LC3/LC3 (microtubule-associated protein 1 light chain 3)-phosphatidylethanolamine conjugate (LC3-II) and SQSTM1 (Fig. 3E and Fig. S3F,G,H). Autophagy block by Baf suppressed clearance of DDF in wild-type but not *Sqstm1* knockout MEFs (Fig. 3E and Fig. S3F,G,H). Together, these data suggest that SQSTM1 is the key mediator of the effect of autophagy on DNA repair kinetics.

### SQSTM1 promotes proteasomal degradation of FLNA and RAD51

**Figure 4.**
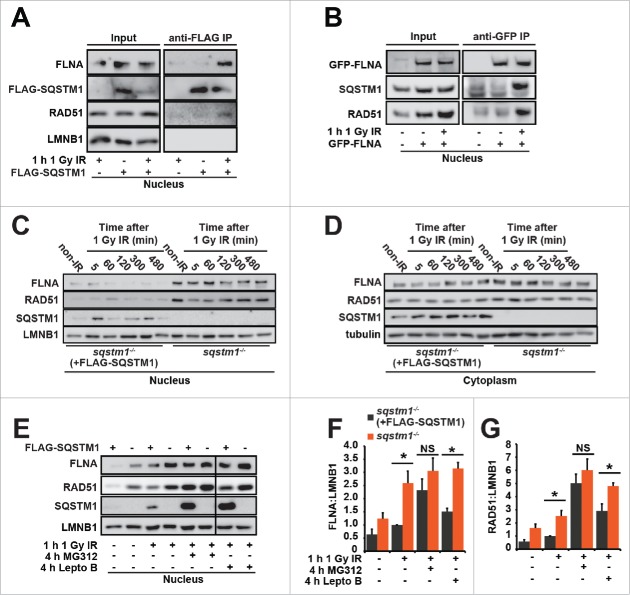
SQSTM1-dependent proteasomal degradation of FLNA and RAD51 regulates DNA repair. (A) *sqstm1*^*−/−*^ MEFs, stably expressing FLAG-SQSTM1 (*sqstm1*^*−/−*^+FLAG-SQSTM1) were irradiated where indicated and 60 min later nuclear fractions were subjected to anti-FLAG IP. The interaction of FLAG-SQSTM1 with endogenous FLNA and RAD51 was detected by immunoblotting. (B) HeLa cells transfected with GFP-FLNA were irradiated, where indicated, and 60 min later nuclear fractions were subjected to anti-GFP IP. The interaction of GFP-FLNA with endogenous SQSTM1 and RAD51 was detected by immunoblotting. (C-D) *sqstm1*^*−/−*^ and *Sqstm1*^*−/−*^+FLAG-SQSTM1 MEFs were irradiated and subjected to cellular fractionation at the time points indicated. Nuclear (C) and cytoplasmic (D) fractions were analyzed for FLNA, RAD51, SSQTM1 and LMNB1 as a loading control. Quantification of blots can be found in Fig. S4B-C. (E-G) *sqstm1*^*−/−*^ and *sqstm1*^*−/−*^+FLAG-SQSTM1 MEFs were pre-incubated with MG132 or leptomycin B (Lepto B) for 3 h where indicated. Cells were irradiated with 1 Gy X-ray irradiation and incubated in the presence of MG132 or leptomycin B for a further 60 min. Nuclear fractions were analyzed for FLNA ((E)and F) and RAD51 ((E)and G) levels and quantified relative to LMNB1. n = 3; error bars represent SEM; NS, not significant; *, p < 0.05; **, p < 0.01; ***, p < 0.001.

To identify the molecular mechanism(s) by which SQSTM1 regulates DNA repair we next immunoprecipitated SQSTM1 from nuclear fractions. A band of approximately 250 kDa was evident specifically in the nuclei from IR-leptomycin B-treated cells (Fig. S4A). Mass spectrometry analyses identified FLNA, previously shown to be required for the recruitment of HR protein RAD51 to the sites of DNA damage and for the efficient clearance of DDF, as the most enriched protein in this sample. In immunoprecipitation assays we could confirm interactions of SQSTM1 with endogenous FLNA and RAD51 and GFP-FLNA with endogenous SQSTM1 and RAD51 taking place specifically in nuclei exposed to IR-induced DNA damage (Fig. 4A,B).^19-21^ Interestingly, nuclear but not cytoplasmic levels of FLNA and RAD51 were found to be increased in *sqstm1*^*−/−*^ MEFs compared to *sqstm1*^*−/−*^ MEFs stably expressing FLAG-SQSTM1 (Fig. 4C,D and Fig. S4B,C). Nuclear levels of SQSTM1 were elevated following IR suggesting its translocation to or retention in the nucleus in response to DNA damage (Fig. 4C).

The detection of increased levels of FLNA and RAD51 in *sqstm1*^*−/−*^ MEFs suggests that SQSTM1 may have a role in the degradation of these proteins. In agreement with our data indicating that nuclear export of SQSTM1 is not required for its effect on DDR (Fig. 2J), treatment with leptomycin B increased basal levels of FLNA and RAD51 but did not cancel the differences between *Sqstm1*^*+/+*^ and *sqstm1*^*−/−*^ MEFs (Fig. 4E-G). In contrast, the proteasomal inhibitor MG132 completely removed the difference in FLNA and RAD51 between the cell lines, suggesting that SQSTM1 plays a role in promoting proteasomal degradation of these proteins (Fig. 4E-G). In agreement with our observations that SQSTM1 acts on DNA repair downstream of autophagy, increased nuclear levels of SQSTM1 in *atg5*^*−/−*^ MEFs compared to wild-type cells following IR correlated with reduced levels of RAD51 (Fig. S4D). In agreement with the above data, treatment with MG132 cancelled the difference in RAD51 levels between *Atg5*^*+/+*^ and *atg5*^*−/−*^ MEFs (Fig. 4E-G and Fig. S4D). Immunofluorescence experiments suggested dynamic interaction between SQSTM1 and RAD51 in nuclear foci (Fig. S4E-H) potentially contributing to the SQSTM1-dependent degradation of RAD51. Indeed, re-expression of SQSTM1 in *sqstm1*^*−/−*^ MEFs reduced the numbers of RAD51 foci following DNA damage (Fig. S5A,B).

**Figure 5.**
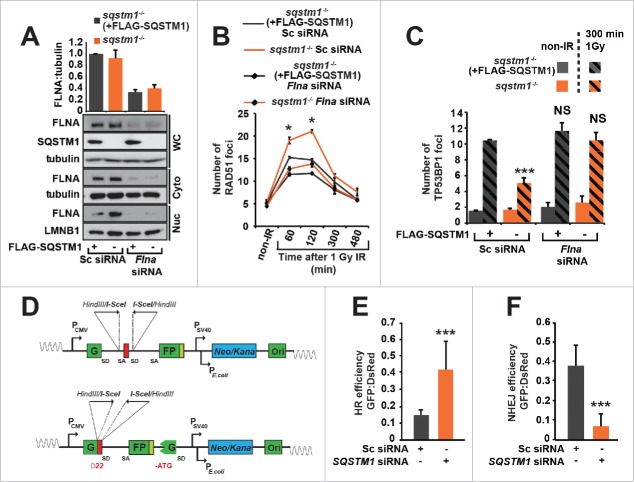
FLNA-dependent effect of SQSTM1 on DNA repair. (A) A representative blot of *Flna* siRNA in *sqstm1*^*−/−*^ and *sqstm1*^*−/−*^+FLAG-SQSTM1 MEFs. (B-C) Quantification of the mean number of RAD51 (p<0.05 at 60 and 120 min when comparing *sqstm1*^*−/−*^ Sc siRNA to all other conditions) (B) and TP53BP1 (C) foci at the indicated times following irradiation in *sqstm1*^*−/−*^ and *sqstm1*^*−/−*^+FLAG-SQSTM1 MEFs treated with control or *Flna* siRNA. (D) Schematic illustration of the HR and NHEJ reporter systems. A *GFP* gene containing a killer intron and I-SceI recognition site integrated into normal human skin fibroblast cells. Upon I-SceI transfection, a DSB is produced that can be repaired by NHEJ or HR leading to production of active GFP. (E) Human skin fibroblasts with integrated HR reporter were transfected with I-SceI plasmid along with the indicated siRNA and analyzed 2 d later by flow cytometry. Quantification of the percentage of GFP vs DsRed is shown. (F) Human skin fibroblasts with integrated NHEJ reporter were transfected with I-SceI plasmid along with the indicated siRNA and analyzed 2 d later by flow cytometry. Quantification of the percentage of GFP vs DsRed is shown. n = 3; error bars represent SEM; NS, not significant; *, p < 0.05; **, p < 0.01; ***, p < 0.001.

Having established that SQSTM1 influences the nuclear levels of FLNA and RAD51, we next investigated whether FLNA mediates the effect of SQSTM1 on DNA repair. Hence, we knocked down FLNA in *sqstm1*^*−/−*^ MEFs with and without stably expressed FLAG-SQSTM1 (Fig. 5A) and examined its effect on RAD51 and TP53BP1 foci following X-ray irradiation. Both re-expression of SQSTM1 in *sqstm1*^*−/−*^ MEFs (which decreased nuclear levels of FLNA, Fig. 5A) and knockdown of *Flna*, reduced the frequency of RAD51-positive DDF (Fig. 5B and Fig. S5C). Therefore, SQSTM1 has an inverse effect on RAD51- and TP53BP1-positive foci (Fig. 2E and Fig. 5B), suggesting that slower resolution of TP53BP1 foci and reduced DNA repair results from an impaired recruitment of RAD51 to the sites of DNA damage. Indeed, knockdown of *Flna* prevented increased clearance of TP53BP1 DDF caused by the loss of SQSTM1 (Fig. 5C and Fig. S5D) suggesting that FLNA also mediates the effect of SQSTM1 on TP53BP1 foci resolution.

Because TP53BP1 and RAD51 promote NHEJ and HR, respectively, we hypothesized that nuclear SQSTM1 facilitates NHEJ at the expense of HR.^10^ In order to test this possibility, we used 2 reporter systems allowing a direct measure of each of these types of DNA repair (Fig. 5D).^22^ In agreement with the above data, knockdown of *SQSTM1* resulted in an increased HR and reduced NHEJ efficiency (Fig. 5E,F).

**Figure 6.**
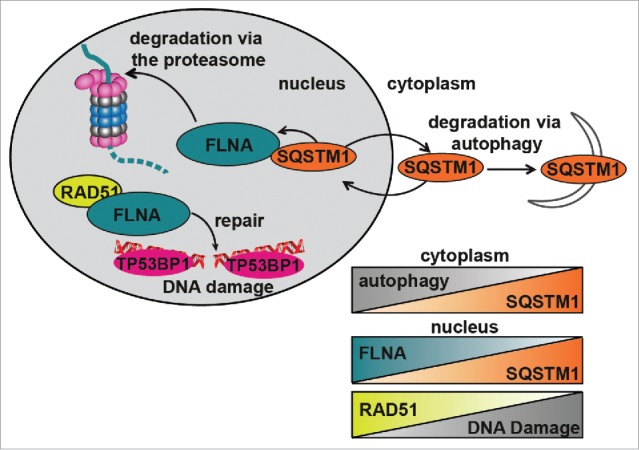
Diagrammatic representation of the role of SQSTM1 in DDR. Following DNA damage, FLNA provides a scaffold to support recruitment of RAD51 and promote DNA repair. We have demonstrated that SQSTM1 can interact with and facilitate the proteasomal degradation of FLNA following DNA damage (induced herein by X-ray irradiation). High levels of nuclear SQSTM1 increased degradation of FLNA, which negatively affected RAD51 recruitment to the sites of DNA damage and therefore increased the amount of time required for DNA damage to be fully resolved. These results have important implications for aging as SQSTM1 levels are carefully regulated by its own turnover via autophagy and proteasomal degradation; perturbation of both have been demonstrated in aging models. Changes in nuclear levels of SQSTM1 could directly contribute to defects in the DDR, further compounding aging-related pathologies. Inverse correlations between autophagy and SQSTM1, nuclear levels of FLNA and SQSTM1 as well as RAD51 and DNA damage are illustrated by the chart.

In conclusion, our data indicate that SQSTM1 suppresses RAD51-mediated HR by promoting proteasomal degradation of FLNA (Fig. 6). This mechanism also underlies the positive role of autophagy on DNA repair as autophagy deficiency, e.g. due to aging, leads to SQSTM1 accumulation impairing the efficiency of HR and reducing the rate and, potentially, quality of DNA repair (Fig. 6).

## Discussion

Although autophagy and the UPS play an important role in the maintenance of a functional proteome and genome, the underlying mechanisms connecting these processes remain poorly understood. Here, we propose a model by which SQSTM1 connects autophagy- and proteasome-mediated protein degradation with DNA repair. Cytoplasmic degradation of SQSTM1 via the autophagy pathway reduces its nuclear levels and, in turn, promotes HR and increases the rate of DDF clearance and DNA repair. Our data indicate that this SQSTM1-dependent mechanism is both necessary and sufficient to mediate the effect of autophagy on DNA repair suggesting that SQSTM1 is the key molecular link between protein and DNA homeostasis.

Interestingly, transport of SQSTM1 from the nucleus to the cytoplasm is not required for its function in the DDR suggesting that it does not act by transporting nuclear proteins for autophagic degradation. We have previously observed that SQSTM1 can mediate proteasomal degradation inside the nucleus and here we identified FLNA and RAD51 as substrates of SQSTM1-dependent proteasomal degradation.^13^ These proteins appear to act downstream of SQSTM1 and mediate the effect of SQSTM1 on DDR. FLNA has previously been implicated in RAD51-dependent DNA repair by promoting the formation of DDF associated with HR.^21^ Our data indicate that SQSTM1-dependent proteasomal degradation of FLNA also results in degradation of RAD51 leading to the suppression of DDR mediated by this protein. Therefore, stabilization of SQSTM1, e.g., due to autophagy impairment, leads to the inhibition of RAD51-dependent DNA repair by HR and accumulation of DDF. The exact mechanism by which SQSTM1 regulates TP53BP1 foci and NHEJ is currently unclear. However, the effect of SQSTM1 on TP53BP1 is also dependent on FLNA suggesting that the changes in NHEJ may be a secondary, compensatory reaction to the changes in HR. Overall, the proposed model (Fig. 6) can explain, at least in part, how autophagy inhibition leads to an accumulation of DNA damage and genomic instability, and contributes to age-related deterioration of cellular function and tumorigenesis.^23,24^

The role of autophagy as a DNA repair-promoting cellular pathway as observed here is in agreement with published reports.^5,6^ Additionally, SQSTM1 has been proposed as a link between autophagy and DDR, although the function of SQSTM1 in DNA repair was unknown and results presented here conceptually extend our understanding of the role of autophagy as a mechanism of genome integrity maintenance.^5^ As an alternative to our identified mechanism linking autophagy and DNA repair pathways, a mechanism involving the degradation of CHEK1 by autophagy has recently been suggested to regulate DNA repair.^4^ We did not observe a significant effect of autophagy perturbations on CHEK1 levels in our experimental conditions (not shown). However, it is important to note that our study used low levels of genotoxic agents to facilitate quantification of individual DDF and thus it is possible that more pronounced changes in signaling pathways downstream of DDR would become evident with more severe DNA damage. Importantly, the careful quantitative analyses outlined in our study clearly demonstrate that autophagic degradation of SQSTM1 is sufficient to explain the role of autophagy in DNA repair processes. Our observations also help to explain how autophagy inhibition may result in an increased dependence on NHEJ at the expense of HR as observed by others.^4^ Indeed, based on the evidence provided here we suggest that autophagy promotes HR by reducing the levels of SQSTM1. Moreover, SQSTM1 is responsible for the net outcome of autophagy perturbation the overall impairment of DNA damage repair. Nevertheless, there are a number of open questions remaining, including about the regulation of SQSTM1 nucleocytoplasmic shuttling in response to DNA damage, which will be a subject of future investigations.

The physiological relevance of our findings is suggested by our observations in aging tissues. Aging is characterized by a gradual impairment of cellular homeostatic pathways such as those involved in protein degradation including autophagy and the UPS (reviewed in refs.^25,26^) Reduced genome stability is another important factor underlying age-related functional decline and contributes to prevalent diseases such as cancer and neurodegeneration. One key question in the field of aging research is how perturbations in protein homeostasis may lead to age-related genomic instability. Our data provide a new mechanistic link between pathways regulating protein and DNA quality control. Autophagy has been proposed to decline during aging, which would consequently lead to the accumulation of SQSTM1, both in the cytoplasmic and, as observed in this report, nuclear compartments. We hypothesize that stabilization of nuclear SQSTM1 in aging tissues would also affect DNA repair mechanisms and prevent efficient clearance of DDF. Nuclear SQSTM1 can also serve as a cell cycle checkpoint protein suppressing proliferation of old cells with an impairment of autophagy. Decreased DNA damage has been shown in the liver following short-term DR, however, the mechanisms are not yet understood.^27^ Our data, suggest that these effects may be mediated, at least in part, by DR-induced autophagic degradation of SQSTM1 thus facilitating DNA damage repair and maintaining a functional genome.

## Materials and methods

### Animals

All mice were inbred C57BL/6 (Harlan, Blackthorn UK). Ethical approval was granted by the LERC Newcastle University, UK. The work was licensed by the UK Home Office (PPL 60/3864) and complied with the guiding principles for the care and use of laboratory animals. Mice were housed in same-sex cages in groups of 4 to 6 (56 × 38 × 18 cm, North Kent Plastics, mouse cage RC1) and individually identified by an ear notch. Mice were housed at 20 ± 2°C under a 12-h light/12-h dark photoperiod with lights on at 7:00 a.m.. The diet used was standard rodent pelleted chow (CRM [P]; Special Diets Services, 801722) for *ad libitum* (AL)-fed mice and the same diet, but as smaller pellets, were offered to dietary restricted (DR) mice. DR mice were offered 60% of AL intake (calculated based on average food intake in 90 control AL mice between 5 and 12 months of age) as one ration at 9:30 a.m. daily. All mice were fed AL until 3 mo of age and then split into AL or DR groups, matched for body mass and food intake.

### Cell culture

Human embryonic lung MRC5 fibroblasts and HeLa cells were obtained from European Collection of Authenticated Cell Cultures (ECACC 05011802 and 93021013) and HEK 293FT were purchased from Life Technologies (R700-07). *Sqstm1* knockout (*sqstm1*^*−/−*^) and wild type (*Sqstm1*^*+/+*^) mouse embryonic fibroblasts (MEFs) were kindly provided by Dr. Eiji Warabi of the University of Tsukuba.^15^ *Atg5*-deficient (*atg5*^*−/−*^) and wild-type (*Atg5*^*+/+*^) MEFs and M5-7 MEFs were kindly provided by Dr Noboru Mizushima (University of Tokyo).^28,29^ All cells were grown in Dulbecco's modified Eagle's medium (Sigma, D6546) supplemented with 10% heat-inactivated fetal bovine serum (Biosera, FB-1001H), 5% penicillin/streptomycin (Invitrogen, 15140122) and 2 mM L-glutamine (Sigma, 59202C) in a humidified atmosphere containing 5% CO_2_ at 37°C. Autophagy was abolished in M5-7 MEFs by treating with 1 μg/ml tetracycline for at least 4 d. HEK 293FT cells were maintained in 500 µg/ml G418 (Sigma, A1720) prior to transfection.

### Drug treatments and X-ray irradiation

Cells were treated with 1 μM etoposide (Sigma, E1383) for 2 h to induce DNA damage, the medium was then replenished (without etoposide) and cells were allowed to recover for 5 h before downstream processing. Cells were incubated with 20 nM leptomycin B (Cell Signaling Technology, 9676) for 1 h to inhibit nuclear protein export. Autophagic flux was inhibited by treatment with bafilomycin A_1_ (Enzo, BML-CM110-0100) at 100 nM for duration of the experiment. Proteasomal inhibition was achieved by treatment with 10 μM MG132 (Sigma, C2211) for 4 h. DNA damage was induced using X-Rad 225 (General Electric) at the doses indicated; media was refreshed immediately after irradiation.

### Plasmids

pEGFP-SQSTM1, pEGFP-ΔPB1SQSTM1, pEGFP-ΔNESSQSTM1 and pEGFP-ΔUBASQSTM1 constructs were previously published.^28^ FLAG-SQSTM1 was kindly provided by Dr. Robert Layfield (University of Nottingham, Nottingham, UK).^13,30,31^ pLKO-puro GFP was kindly provided by Dr. Simon Wilkinson (Edinburgh Cancer Research Center, University of Edinburgh). pG-AcGFP-53BP1c has been described previously.^32^

### Cloning

For lentiviral expression full-length wild-type FLAG-tagged SQSTM1 was subcloned into the pLenti6-UbC/V5-DEST vector (ThemoFisher Scientific, V49910) using EcoRI and XhoI (New England BioLabs, R0101, R0146). Briefly, FLAG-SQSTM1 and pLENTI6/V5-DEST vector were digested with EcoRI and XhoI prior to gel purification using Qiaquick GEL Extraction Kit (Qiagen, 28704). The vector was dephosphorylated by calf intestinal alkaline phosphatase (Fermentas, EF0651) and the ligation with FLAG-SQSTM1 was carried out using T4 DNA Ligase (New England BioLabs, M0202S). mCherry-53BP1c for lentiviral expression was generated as follows: a 2.7-kb C-terminal portion of *TP53BP1* (53BP1c), was excised from pAcGFP-53BP1c,^33^ using BamHI and XhoI and ligated into pENTR2B (Invitrogen, A10463) to create pENTR2B-53BP1c. The sequence for mCherry fluorescent protein was amplified via PCR from pRSETB-mCherry,^34^ incorporating SalI sites at both ends and a 5 amino acid linker at the 3′ end.^32^ This product was ligated into pENTR2B-53BP1c SalI site in frame 5′ of 53BP1c. A correct, sequence-verified clone was then recombined into pLenti6-UbC/V5-DEST using LR Clonase following the manufacturer's instructions (Invitrogen, 11791100) to produce pLenti6-mCherry-53BP1c.

### Transformations

All bacterial transformations for transiently expressed plasmids was performed using α-select GOLD Efficiency chemically competent cells (Bioline, BIO-85027) and grown overnight at 37°C. Bacteria transformations for lentiviral plasmids was performed in NEB stable Competent *E. coli* (High Efficiency; New England BioLabs, C3040H) and grown at 30°C for 24 h. DNA was purified using PureYield Plasmid Midiprep System (Promega, A2492).

### Transfections

Cells were transfected using polyethylenimine (PEI) (Sigma, 408727) as described in Shaner et al or Lipofectamine 2000 (Life Technologies, 11668) according to the manufacturer's protocols for 24 h prior to lysis or fixation.^34^

### Lentiviral transductions

Lentivirus particles were generated in HEK293FT following the manufacturer's protocol (Life Technologies, R7007). *GFP* and *Sqstm1* shRNA (Dharmacon, RHS4459 and TRCN0000098618) was stably introduced into MRC5 and FLAG-SQSTM1 was stably re-introduced into *sqstm1*^−/−^ MEFs through lentiviral transduction. HEK 293FT cells were seeded in antibiotic-free medium supplemented with 0.1 mM MEM nonessential amino acids (Thermo Fisher Scientific, 11140050) and then cotransfected with either lentiviral expression vectors and 2^nd^ generation (for pKLO shRNA vectors) or 3^rd^ generation (for pLenti vectors) packaging system plasmids (Thermo Fisher Scientific, K497500). After 24 h, media was replaced with fresh media without antibiotics. Forty-eight h after transfection, viral transduction was performed by transferring media from HEK 293FT cells to 70% confluent MRC5 or *Sqstm1* knockout (*sqstm1*^−/−^) MEFs in the presence of 6 μg/ml Polybrene (Sigma, H9268). Media containing virus was replaced after 24 h with fresh media containing 8 μg/ml of blasticidin (Invitrogen, A1113903) for selection of transduced cells. Media was replaced every 2-3 d for 10-12 d by keeping the antibiotic selection. Transduced MEFs were then maintained in lower levels of blasticidin (4 μg/ml) until seeding for experimental purposes. Transduced MRC5s were then maintained in lower levels of puromycin (Gibco, A1113803; 0.5 μg/ml) until seeding for experimental purposes

### siRNA

ON-TARGETplus SMARTpool siRNA against mouse *Sqstm1* (L-047628-01), *Flna* (L-058520-01) and nontargeting SMARTpool siRNA (D-001810-04) were purchased from Dharmacon. Final siRNA concentrations of 100 nM were used for 96 h for silencing, and transfections were carried out using Lipofectamine 2000 as per company instructions.

### Immunofluorescence

Cells were grown in 12- or 24-well plates on sterile cover slips (30,000 cells/ml 48 h). Following treatment, cells were fixed for 8 min in 2% formaldehyde, permeabilized and blocked in PBG-Triton buffer (0.5% BSA [Sigma, 05482], 0.2% cold water fish skin gelatin (Sigma, G7765), 0.5% Triton X-100 (Sigma, X100) in phosphate-buffered saline [PBS; Cell Signaling Technology, 9808]) for 45 min. Cells were incubated with primary antibody: guinea pig SQSTM1 (Progen, GP-62-C; 1:200), rabbit TP53BP1 (Cell Signaling Technology, 4937, 1:200), rabbit RAD51 (Milipore, ABE257; 1:500), in PBG-Triton buffer at 4°C overnight, washed 3 times in PBG-Triton and incubated with appropriate secondary antibodies conjugated to Alexa Fluor® 488/594/647 (Invitrogen, A-11029, A-11020, A-21237, A-11034, A-11037, A21244, A-11076; 1:2000) in PBG-Triton for 1 h at room temperature. Cells were then washed 3 times in PBS and mounted using ProLong Gold (Life Technologies, P3690).

Paraffin sections were deparaffinized with Histoclear (Fisher Scientific, HS-202-1GAL) and ethanol, and antigen was retrieved by incubation in 0.01 M citrate buffer, pH 6.0 at 95°C for 20 min. Slides were incubated in 0.9% H_2_O_2_ for 30 min and subsequently placed in blocking buffer (0.1% BSA in PBS, including 5% normal goat serum (Vector Lab, S1000]) for 30–60 min at room temperature. Livers were further blocked with Avidin/Biotin (Vector Lab, SP-2001) for 15 min each. Primary antibody (rabbit γH2AFX 1:200 in blocking buffer; Cell Signaling Technology, 9718,) was applied overnight at 4°C. Slides were washed 3 times with PBS and incubated for 30 min with secondary antibody (Vector Lab, PK-4001). Slides were washed 3 times with PBS and incubated with Fluorescein Avidin DCS (Vector Lab, A-2011; 1:500 in PBS), which was applied for 20 min. Slides were washed 3 times with PBS and incubated for 30 min with blocking buffer. Second primary antibody (guinea pig SQSTM1; Progen, GP-62-C; 1:100 in blocking buffer) was applied overnight at 4°C. Slides were washed 3 times with PBS and incubated for 30 min with secondary antibody (anti-guinea pig Alexa Fluor® 594; Invitrogen, A-11076; 1:2000 in blocking buffer). Sections were stained with DAPI for 5-10 min and mounted in Vectashield mounting medium (Vector Lab, H-1200).

Cells were imaged with a Leica DM 5500B Widefield Microscope through an HCX PL APO 100x/1.40-0.70 or oil HCX PL APO 40x/1.25 oil objective using a Leica DFC 360 FX camera. Alternatively, for colocalization analysis and live cell imaging analysis, images were captured using a Zeiss CellObsever spinning disk confocal microscope equipped with: CSUX1 spinning disk confocal head (Yokogawa), and Quant EM CCD (Photometrics), using a 405, 488 and 561 nm lasers and 63× 1.4NA objective (Zeiss) driven by Axiovision software (v4.8.1, Zeiss, Cambridge, UK).

### EdU incorporation

EdU incorporation was performed for 24 h prior to the detection with the Click-IT EdU Alexa Fluor 594 Imaging Kit (Invitrogen, C103339) following the manufacturer's instructions.

### Cellular fractionations

Cellular fractionation was carried out as in ref.^35^ Briefly, 6 × 10^5^ cells were seeded on 10-cm dishes 48 h prior to collection. Cells were washed in ice-cold PBS, scraped in 1 ml ice-cold PBS and centrifuged for 10 sec at 17,900g at 4°C. The supernatant fraction was aspirated and cells were resuspended (triturated 5 times) in 1 ml ice-cold 0.1% NP40 (Sigma, 18896) in PBS. 200 µL was collected in a fresh tube (whole cell sample). Samples were centrifuged again for 10 sec at 17,900g at 4°C and the supernatant fraction, which represents the cytoplasmic fraction, was collected in a fresh tube. The pellet was resuspended (triturated once) in 1 ml ice-cold 0.1% NP40 in PBS. Samples were centrifuged for 10 sec at 17,900g at 4°C, the supernatant fraction was discarded and the nuclear pellet fraction was processed as described below.

For immunoblot: Whole cell samples and cytoplasmic fractions were mixed 3:1 with 4x Laemmli sample buffer (Bio-Rad, 161-0747), sonicated using a microprobe for 5 sec on ice and boiled at 100°C for 5 min in the presence of 2.5% β-mercaptoethanol (β-ME; Sigma, M6250). Nuclear pellets were resuspended in 200 µL 1x Laemmli sample buffer, sonicated using a microprobe for 5 sec on ice and boiled at 100°C for 5 min in the presence of 2.5% β-ME then centrifuged for 10 min at 17,900g at 4°C and transferred to a new tube.

For immunoprecipitation: The nuclear pellet fractions were resuspended in 200 µL IP buffer (50 mM Tris, pH 7.5, 150 mM NaCl, 2 mM MgCl_2_, 1 mM CaCl_2_, 0.1% Tween 20 (Sigma, P1379), 0.5% Triton-X100 and 2x Halt Protease & Phosphatase inhibitor cocktail [Thermo Scientific, 1861280]). The samples were sonicated using a microprobe for 5 sec on ice and boiled at 100°C for 5 min in the presence of 2.5% β-ME. Samples were then centrifuged for 10 min at 17,900g at 4°C and transferred to a new tube. The immunoprecipitation protocol was then preformed as described below.

### Immunoprecipitation

Cells were seeded at 6 × 10^5^ in a 10-cm dish and transfected as described above 24 h later. Following another 24 h, nuclear fractions were prepared as described above.

For FLAG-tagged protein: Lysates were incubated with prewashed and equilibrated anti-FLAG M2 magnetic beads (Sigma, M8823) for 2 h at 4°C with constant rotation. Beads were washed twice with lysis buffer and the affinity-isolated protein was eluted from the beads by incubation with 25 µl 0.2 M glycine-HCl, pH 2.5, for 10 min at room temperature. Eluent was neutralized by the addition of 2.5 µl Tris-HCl, pH 8.8. The samples were then mixed with sample buffer and boiled at 100°C for 5 min in the presence of 2.5% β-ME before being subjected to SDS-PAGE and immunoblot.

For GFP-tagged protein: Lysates were incubated with 3 µl anti-GFP rabbit serum (Life Technologies, A-6455) for 1 h at 4°C with constant rotation. Lysates were then incubated with 20 µl pre-washed protein A Sepharose beads (Generon, PC-A5) for 1 h at 4°C with constant rotation. Beads were then washed twice in IP buffer, mixed with sample buffer and boiled at 100°C for 5 min in the presence of 2.5% β-ME. Samples were centrifuged for 10 min at 17,900g at 4°C and transferred to a new tube. Samples were then subjected to SDS-PAGE and immunoblot.

To demonstrate the levels of proteins in cell lysates used for immunoprecipitation, 5% of cell lysates were loaded per lane and indicated as “Input.”

### Immunoblotting

Cells were seeded in 6-well plates 48 h prior to treatments. After treatments, cells were washed in ice-cold PBS and then lysed in RIPA buffer (150 mM NaCl, 1% NP40, 0.5% sodium deoxycholate (Sigma, D6750) 0.1% SDS (ThermoFisher Scientific, AM9820), 50 mM Tris, pH 7.4, supplemented with 2x Halt Protease & Phosphatase inhibitor cocktail). Cell lysates were centrifuged at 4°C at 17,900g for 10 min to remove insoluble cellular components. Protein concentration was measured using the DC Protein Assay Kit I (Bio-Rad, 500-0112) and a FLUOstar Omega plate reader (BMG Labtech). Samples were prepared by diluting in SDS-Loading buffer (Bio-Rad, 161-0747) and boiled at 100°C for 5 min in the presence of 2.5% β-ME. Protein (20-30 μg) was analyzed by SDS-PAGE and western blot; briefly, samples were separated on 5–15% Tris-glycine gels and transferred to Immobilon®-P (Millipore, IPVH00010) membrane using a Trans-Blot® Semi-Dry transfer apparatus (Bio-Rad). Blots were incubated with a blocking solution (PBS containing 5% fat-free milk, 0.1% Tween 20) for 1 h at room temperature. Blots were incubated with primary antibodies diluted in blocking solution at 4°C overnight. The following primary antibodies were used: rabbit ATG5 (Sigma, A0856; 1:1000), mouse FLAG M2 (Sigma, F1804; 1:2000), rabbit GAPDH (Cell Signaling Technology, 5174; 1:1000), rabbit LC3B (Cell Signaling Technology, 3868; 1:1000), guinea pig SQSTM1 (Progen, GP-62-C; 1:1000), rabbit RAD51 (Millipore, ABE257; 1:1000), rabbit FLNA (Cell Signaling Technology, 4762; 1:1000), mouse α-tubulin (Developmental Studies Hybridoma Bank, 12G10; 1:10,000), rabbit LMNB1 (lamin B1; Abcam, ab16048; 1:5000). Blots were incubated with appropriate secondary antibodies conjugated to horseradish peroxidase (anti-guinea pig: Dako, P0141; anti-mouse: Sigma, A2554; anti-rabbit: Sigma, A0545) for 1 h at room temperature. Clarity western ECL substrate (Bio-Rad, 170-5061) was used to visualize chemiluminescence on LAS4000 (Fujifilm). Quantification of blots was carried out using ImageJ (v1.45j, http://rsb.info.nih.gov/ij/).

### Neutral comet assays

Cells were trypsinized and frozen in 10% DMSO in fetal bovine serum and stored at −80°C prior to downstream processing. Cells were washed in cold PBS and resuspended in 0.7% Low Melting Point agarose (Sigma, A9414) at 37°C to a concentration of 2 × 10^5^ cells/ml. Cell/agarose mix (70 μl) was placed on slides previously coated in 1% agarose. Slides were incubated in lysis buffer (2.5 M NaCl, 100 nM EDTA, 10 nM Tris, pH 10, 250 nM NaOH, 10% DMSO, 1% Triton X-100) for 1 h at 4°C. Slides were then washed twice in cold PBS.

Samples were subjected to electrophoresis for 30 min at 25 V at 4°C in Tris-borate EDTA buffer (Sigma, T3913). Slides were then washed twice in cold PBS and stained with Sybr Gold (Life Technologies, S11494) in Tris-borate EDTA buffer for 45 min. Slides were washed twice in MilliQ water and allowed to dry. Samples were imaged using an Olympus BX51 widefield microscope with Olympus UPlanFL 20x/0.50 air objective. Comets were scored using Comet assay IV (Perceptive Instruments). For each sample, 100 randomly captured comets (50 cells on each of 2 comet slides) were quantified.

### Time-lapse imaging of TP53BP1 DNA damage foci

*sqstm1*^*−/−*^ and *Sqstm1*^*+/+*^ MEFs, stably expressing mCherry-53BP1c were seeded on a 35-mm glass bottomed dish (ThermoFisher Scientific, 150680) 48 h prior to treatment. Cells were irradiated with 0.25 Gy X-ray irradiation and immediately transferred to the heated, XLmulti S1 humidified stage (95% air, 5% CO_2_) of a Zeiss CellObsever spinning disk confocal for imaging. Images were captured using a 561-nm laser and 40 × 1.3NA objective (Zeiss) driven by Axiovision software (v4.8.1, Zeiss, Cambridge, UK). Z-stacks encompassing the entire cell were taken every 10 min for 8 h. Maximum projections were created and the number of TP53BP1 foci were tracked manually using ImageJ (NIH v1.45j, http://rsb.info.nih.gov/ij/).

### Mass spectrometry analyses

Excised bands were digested in-gel and the resulting tryptic peptides analyzed by LC-MSMS using an Orbitrap XL (Themo Scientific) coupled to a nanoAcquity (Waters). MSMS data were acquired in a top 6 DDA fashion and raw files were processed in Proteome Discover v1.4 using the Sequest search engine against a Uniprot human database (downloaded 030314, 68,710 entries). CAM cysteine was set as a fixed modification with oxidized methionine and deamidated asparagine-glutamine as potential variable modifications. FDR calculations were performed using Percolator with peptides filtered to 0.01 FDR.

### NHEJ and HR reporter assays

NHEJ and HR reporter assays were carried out as described in refs. 36–38. Briefly, normal human skin fibroblasts (HCA2) immortalized by hTERT (from Dr. J. Campisi, The Buck Institute for Research on Aging, USA) carrying chromosomally integrated NHEJ and HR reporters^38^ were transfected with 20 µM of the indicated siRNA for 48 h using Amaxa Nucleofector (T20 program). Forty-eight h after the second round of transfection with a mixture of 3 μg I-SceI-expressing plasmid (Addgene, 26477; deposited by Dr. Maria Jasin), 0.1 μg pDsRed2-N1 (Clontech, 632406) and 20 µM of the indicated siRNA, cells were harvested and analyzed by flow cytometry.^36-38^

### Quantification and statistical analysis

Quantifications were performed by blind scoring of slides as described previously.^25^ A constant threshold was applied to all the images in the z-stack, and for every image within each experiment. Following application of the colocalization plug-in, all channels were projected (max) and quantified using the Analyze particle plugin (particles 5 pixels and above were included). Quantification was carried out on 30-50 cells per condition. Quantification of immunoblots was carried out using ImageJ software (NIH v1.45j, http://rsb.info.nih.gov/ij/). Two-tailed, paired or unpaired Student *t* tests were carried out on experimental data from at least 3 individual experiments using Excel. A one-way Anova was used for multiple comparisons between groups using Sigma Plot.

## Supplementary Material

1210368_Supplemental_Material.zip

## Abbreviations

AL: *ad libitum*
ATG5: autophagy-related 5
ATG7: autophagy-related 7
ATM: ATM serine-threonine kinase
ATR: ATR serine/threonine kinase
Baf: bafilomycin A_1_
CHEK1: checkpoint kinase 1
CMA: chaperone-mediated autophagy
DDF: DNA damage foci
DDR: DNA damage response
DR: dietary restricted
EdU: 5-ethynyl-2′-deoxyuridine
FLNA: filamin A
GAPDH: glyceraldehyde-3-phosphate dehydrogenase
GFP: green fluorescent protein
γH2AFX: H2A histone family member X; phosphorylated
HPRT1: hypoxanthine phosphoribosyltransferase 1
IR: X-ray irradiation
MAP1LC3A-II/LC3A-II: microtubule-associated protein 1 light chain 3 α; phosphatidylethanolamine conjugate
NES: nuclear export signal
NHEJ: nonhomologous end joining
β-ME: β-mercaptoethanol
MEFs: mouse embryonic fibroblasts
PB1: Phox and Bem1 domain
RAD51: RAD51 recombinase
RB1CC1/FIP200: RB1 inducible coiled-coil 1
SQSTM1/p62: sequestosome 1
TP53BP1: tumor protein p53 binding protein 1
UBA: ubiquitin-associated domain
UPS: ubiquitin-proteasome system

## References

[1] Jackson SP, Bartek J The DNA-damage response in human biology and disease. Nature 2009; 461:1071-8, doi:http://www.nature.com/nature/journal/v461/n7267/suppinfo/nature08467_S1.html; PMID:19847258; http://dx.doi.org/10.1038/nature08467 19847258 PMC2906700

[2] Mehta A, Haber JE Sources of DNA double-strand breaks and models of recombinational DNA repair. Cold Spring Harb Perspect Biol 2014; 6:a016428, doi:10.1101/cshperspect.a016428; PMID:25104768; http://dx.doi.org/10.1101/cshperspect.a016428 25104768 PMC4142968

[3] Krogan NJ, Lam MH, Fillingham J, Keogh MC, Gebbia M, Li J, Datta N, Cagney G, Buratowski S, Emili A, et al. Proteasome involvement in the repair of DNA double-strand breaks. Mol Cell 2004; 16:1027-34, doi:10.1016/j.molcel.2004.11.033; PMID:15610744; http://dx.doi.org/10.1016/j.molcel.2004.11.033 15610744

[4] Liu EY, Xu N, O'Prey J, Lao LY, Joshi S, Long JS, O'Prey M, Croft DR, Beaumatin F, Baudot AD, et al. Loss of autophagy causes a synthetic lethal deficiency in DNA repair. Proc Natl Acad Sci U S A 2015; 112:773-8, doi:10.1073/pnas.1409563112; PMID:25568088; http://dx.doi.org/10.1073/pnas.1409563112 25568088 PMC4311830

[5] Bae H, Guan JL Suppression of autophagy by FIP200 deletion impairs DNA damage repair and increases cell death upon treatments with anticancer agents. Mol Cancer Res 2011; 9:1232-41, doi:10.1158/1541-7786.MCR-11-0098; PMID:21807966; http://dx.doi.org/10.1158/1541-7786.MCR-11-0098 21807966 PMC3175275

[6] Robert T, Vanoli F, Chiolo I, Shubassi G, Bernstein KA, Rothstein R, Botrugno OA, Parazzoli D, Oldani A, Minucci S, et al. HDACs link the DNA damage response, processing of double-strand breaks and autophagy. Nature 2011; 471:74-9, doi:http://www.nature.com/nature/journal/v471/n7336/abs/10.1038-nature09803-unlocked.html-supplementary-information; PMID:21368826; http://dx.doi.org/10.1038/nature09803 21368826 PMC3935290

[7] Ivanov A, Pawlikowski J, Manoharan I, van Tuyn J, Nelson DM, Rai TS, Shah PP, Hewitt G, Korolchuk VI, Passos JF, et al. Lysosome-mediated processing of chromatin in senescence. J Cell Biol 2013; 202:129-43, doi:10.1083/jcb.201212110; PMID:23816621; http://dx.doi.org/10.1083/jcb.201212110 23816621 PMC3704985

[8] Rello-Varona S, Lissa D, Shen S, Niso-Santano M, Senovilla L, Mariño G, Vitale I, Jemaá M, Harper F, Pierron G, et al. Autophagic removal of micronuclei. Cell Cycle 2012; 11:170-6, doi:10.4161/cc.11.1.18564; PMID:22185757; http://dx.doi.org/10.4161/cc.11.1.18564 22185757

[9] Park C, Suh Y, Cuervo AM Regulated degradation of Chk1 by chaperone-mediated autophagy in response to DNA damage. Nat Commun 2015; 6:6823, doi:10.1038/ncomms7823; PMID:25880015; http://dx.doi.org/10.1038/ncomms7823 25880015 PMC4400843

[10] Bunting SF, Callén E, Wong N, Chen HT, Polato F, Gunn A, Bothmer A, Feldhahn N, Fernandez-Capetillo O, Cao L, et al. 53BP1 inhibits homologous recombination in Brca1-deficient cells by blocking resection of DNA breaks. Cell 2010; 141:243-54, doi:10.1016/j.cell.2010.03.012; PMID:20362325; http://dx.doi.org/10.1016/j.cell.2010.03.012 20362325 PMC2857570

[11] Korolchuk VI, Menzies FM, Rubinsztein DC Mechanisms of cross-talk between the ubiquitin-proteasome and autophagy-lysosome systems. FEBS Lett 2010; 584:1393-8, doi:; http://dx.doi.org/10.1016/j.febslet.2009.12.047; PMID:20040365; http://dx.doi.org/10.1016/j.febslet.2009.12.047 20040365

[12] Korolchuk VI, Menzies Fm Fau - Rubinsztein DC, Rubinsztein DC A novel link between autophagy and the ubiquitin-proteasome system. Autophagy 2009; 5:862-3; PMID:19458478; http://dx.doi.org/10.4161/auto.8840 19458478

[13] Pankiv S, Lamark T, Bruun JA, Øvervatn A, Bjørkøy G, Johansen T Nucleocytoplasmic Shuttling of p62/SQSTM1 and Its Role in Recruitment of Nuclear Polyubiquitinated Proteins to Promyelocytic Leukemia Bodies. J Biol Chem 2010; 285:5941-53, doi:10.1074/jbc.M109.039925; PMID:20018885; http://dx.doi.org/10.1074/jbc.M109.039925 20018885 PMC2820819

[14] Tsabar M, Eapen VV, Mason JM, Memisoglu G, Waterman DP, Long MJ, Bishop DK, Haber JE Caffeine impairs resection during DNA break repair by reducing the levels of nucleases Sae2 and Dna2. Nucleic Acids Res 2015; 43:6889-901, doi:10.1093/nar/gkv520; PMID:26019182; http://dx.doi.org/10.1093/nar/gkv520 26019182 PMC4538808

[15] Komatsu M, Waguri S, Koike M, Sou YS, Ueno T, Hara T, Mizushima N, Iwata J, Ezaki J, Murata S, et al. Homeostatic levels of p62 control cytoplasmic inclusion body formation in autophagy-deficient mice. Cell 2007; 131:1149-63, doi:10.1016/j.cell.2007.10.035; PMID:18083104; http://dx.doi.org/10.1016/j.cell.2007.10.035 18083104

[16] Du J, Gebicki JM Proteins are major initial cell targets of hydroxyl free radicals. Int J Biochem Cell Biol 2004; 36:2334-43, doi:10.1016/j.biocel.2004.05.012; PMID:15313477; http://dx.doi.org/10.1016/j.biocel.2004.05.012 15313477

[17] Liao W, McNutt MA, Zhu WG The comet assay: a sensitive method for detecting DNA damage in individual cells. Methods 2009; 48:46-53, doi:10.1016/j.ymeth.2009.02.016; PMID:19269328; http://dx.doi.org/10.1016/j.ymeth.2009.02.016 19269328

[18] Johansen T, Lamark T Selective autophagy mediated by autophagic adapter proteins. Autophagy 2011; 7:279-96, doi:10.4161/auto.7.3.14487; PMID:21189453; http://dx.doi.org/10.4161/auto.7.3.14487 21189453 PMC3060413

[19] Meng X, Yuan Y, Maestas A, Shen Z Recovery from DNA damage-induced G2 arrest requires actin-binding protein filamin-A/Actin-binding protein 280. J Biol Chem 2004; 279:6098-105, doi:10.1074/jbc.M306794200; PMID:14660646; http://dx.doi.org/10.1074/jbc.M306794200 14660646

[20] Yue J, Lu H, Liu J, Berwick M, Shen Z Filamin-A as a marker and target for DNA damage based cancer therapy. DNA Repair 2012; 11:192-200; PMID:22051193; http://dx.doi.org/10.1016/j.dnarep.2011.10.019 22051193 PMC3267325

[21] Yue J, Wang Q, Lu H, Brenneman M, Fan F, Shen Z The cytoskeleton protein filamin-A is required for an efficient recombinational DNA double strand break repair. Cancer Res 2009; 69:7978-85, doi:D - NLM: NIHMS142043. D - NLM: PMC2763018 EDAT- 2009/10/0806:00MHDA-2009/12/1606:00 CRDT- 2009/10/08 06:00 PHST- 2009/10/06 [aheadofprint] AID - 0008-5472.CAN-09-2177 [pii] AID - 10.1158/0008-5472.CAN-09-2177 [doi] PST - ppublish; PMID:19808958; http://dx.doi.org/10.1158/0008-5472.CAN-09-2177 19808958 PMC2763018

[22] Seluanov A, Mao Z, Gorbunova V Analysis of DNA Double-strand Break (DSB) repair in mammalian cells. J Vis Exp 2010; 144-50; http://dx.doi.org/10.3791/2002 PMC3157866 20864925

[23] Mathew R, Karp CM, Beaudoin B, Vuong N, Chen G, Chen HY, Bray K, Reddy A, Bhanot G, Gelinas C, et al. Autophagy suppresses tumorigenesis through elimination of p62. Cell 2009; 137:1062-75, doi:10.1016/j.cell.2009.03.048; PMID:19524509; http://dx.doi.org/10.1016/j.cell.2009.03.048 19524509 PMC2802318

[24] Vessoni AT, Filippi-Chiela EC, Menck CF, Lenz G Autophagy and genomic integrity. Cell Death Differ 2013; 20:1444-54, doi:10.1038/cdd.2013.103; PMID:23933813; http://dx.doi.org/10.1038/cdd.2013.103 23933813 PMC3792426

[25] Korolchuk VI, Saiki S, Lichtenberg M, Siddiqi FH, Roberts EA, Imarisio S, Jahreiss L, Sarkar S, Futter M, Menzies FM, et al. Lysosomal positioning coordinates cellular nutrient responses. Nat Cell Biol 2011; 13:453-60, doi:10.1038/ncb2204; PMID:21394080; http://dx.doi.org/10.1038/ncb2204 21394080 PMC3071334

[26] Saez I, Vilchez D The mechanistic links between proteasome activity, aging and age-related diseases. Curr Genomics 2014; 15:38-51, doi:10.2174/138920291501140306113344; PMID:24653662; http://dx.doi.org/10.2174/138920291501140306113344 24653662 PMC3958958

[27] Wang C, Maddick M, Miwa S, Jurk D, Czapiewski R, Saretzki G, Langie SA, Godschalk RW, Cameron K, von Zglinicki T Adult-onset, short-term dietary restriction reduces cell senescence in mice. Aging (Albany NY) 2010; 2:555-66, doi:D - NLM: PMC2984605 EDAT- 2010/09/17 06:00 MHDA- 2011/01/12 06:00 CRDT- 2010/09/17 06:00 AID - 100196 [pii] PST - ppublish; PMID:20844316; http://dx.doi.org/10.18632/aging.100196 20844316 PMC2984605

[28] Kuma A, Hatano M, Matsui M, Yamamoto A, Nakaya H, Yoshimori T, Ohsumi Y, Tokuhisa T, Mizushima N The role of autophagy during the early neonatal starvation period. Nature 2004; 432:1032-6, doi:10.1038/nature03029; PMID:15525940; http://dx.doi.org/10.1038/nature03029 15525940

[29] Hosokawa N, Hara Y, Mizushima N Generation of cell lines with tetracycline-regulated autophagy and a role for autophagy in controlling cell size. FEBS Lett 2007; 581:2623-9, doi:10.1016/j.febslet.2007.05.061; PMID:17553497 17553497

[30] Bjorkoy G, Lamark T, Brech A, Outzen H, Perander M, Overvatn A, Stenmark H, Johansen T p62/SQSTM1 forms protein aggregates degraded by autophagy and has a protective effect on huntingtin-induced cell death. J Cell Biol 2005; 171:603-14, doi:10.1083/jcb.200507002; PMID:16286508; http://dx.doi.org/10.1083/jcb.200507002 16286508 PMC2171557

[31] Lamark T, Perander M, Outzen H, Kristiansen K, Øvervatn A, Michaelsen E, Bjørkøy G, Johansen T Interaction codes within the family of mammalian Phox and Bem1p domain-containing proteins. J Biol Chem 2003; 278:34568-81, doi:10.1074/jbc.M303221200; PMID:12813044; http://dx.doi.org/10.1074/jbc.M303221200 12813044

[32] Nelson G, Buhmann M, von Zglinicki T DNA damage foci in mitosis are devoid of 53BP1. Cell Cycle 2009; 8:3379-83, doi:10.4161/cc.8.20.9857; PMID:19806024; http://dx.doi.org/10.4161/cc.8.20.9857 19806024

[33] Najat D, Garner T, Hagen T, Shaw B, Sheppard PW, Falchetti A, Marini F, Brandi ML, Long JE, Cavey JR, et al. Characterization of a non-UBA domain missense mutation of sequestosome 1 (SQSTM1) in Paget's disease of bone. J Bone Miner Res 2009; 24:632-42, doi:10.1359/jbmr.081204; PMID:19049332; http://dx.doi.org/10.1359/jbmr.081204 19049332

[34] Shaner NC, Campbell RE, Steinbach PA, Giepmans BN, Palmer AE, Tsien RY Improved monomeric red, orange and yellow fluorescent proteins derived from Discosoma sp. red fluorescent protein. Nat Biotech 2004; 22:1567-72, doi:doi:http://www.nature.com/nbt/journal/v22/n12/suppinfo/nbt1037_S1.html; http://dx.doi.org/10.1038/nbt1037 15558047

[35] Suzuki K, Bose P, Leong-Quong RY, Fujita DJ, Riabowol K REAP: A two minute cell fractionation method. BMC Res Notes 2010; 3:294, doi:10.1186/1756-0500-3-294; PMID:21067583; http://dx.doi.org/10.1186/1756-0500-3-294 21067583 PMC2993727

[36] Mao Z, Bozzella M, Seluanov A, Gorbunova V Comparison of nonhomologous end joining and homologous recombination in human cells. DNA Repair 2008; 7:1765-71, doi:10.1016/j.dnarep.2008.06.018; PMID:18675941; http://dx.doi.org/10.1016/j.dnarep.2008.06.018 18675941 PMC2695993

[37] Mao Z, Seluanov A, Fau-, Jiang Y, Jiang Y, Fau-, Gorbunova V, Gorbunova V TRF2 is required for repair of nontelomeric DNA double-strand breaks by homologous recombination. doi:D - NLM: PMC1941808 EDAT- 2007/08/03 09:00 MHDA- 2007/09/21 09:00 CRDT- 2007/08/03 09:00 PHST- 2007/08/01 [aheadofprint] AID - 0702410104 [pii] AID - 10.1073/pnas.0702410104 [doi] PST - ppublish PMC1941808 17670947

[38] Seluanov A, Mittelman D, Pereira-Smith OM, Wilson JH, Gorbunova V DNA end joining becomes less efficient and more error-prone during cellular senescence. Proc Natl Acad Sci U S A 2004; 101:7624-9, doi:10.1073/pnas.0400726101; PMID:15123826; http://dx.doi.org/10.1073/pnas.0400726101 15123826 PMC419656

