# Supplementary material for: SQSTM1/p62 mediates crosstalk between autophagy and the UPS in DNA repair
Source: Autophagy. 2016 Jul 8;12(10):1917–30. doi: 10.1080/15548627.2016.1210368 (PMC5391493; doi:10.1080/15548627.2016.1210368)
Supplement: 1210368_Supplemental_Material.zip [file kaup-12-10-1210368-s001.zip › 1210368_Supplemental Material.docx]

**Supplemental Information**

**SQSTM1/p62 mediates crosstalk between autophagy and the UPS in DNA repair**

Graeme Hewitt, Bernadette Carroll, Rezazadeh Sarallah, Clara Correia‐Melo, Mikołaj Ogrodnik, Glyn Nelson, Elsje G. Otten, Diego Manni, Robin Antrobus, Brian A. Morgan, Thomas von Zglinicki, Diana Jurk, Andrei Seluanov, Vera Gorbunova, Terje Johansen, João F. Passos & Viktor I. Korolchuk

**Supplementary Figures:**

**Figure S1.** Nuclear SQSTM1 colocalization with DDF increases with age *in vivo* and is reduced by DR. (**A**) Representative images of γH2AFX and TP53BP1 in MRC5 human fibroblasts with and without 1 Gy X-ray irradiation. (**B**) Representative blot of ATM in AT cells with and without 1 Gy X-ray irradiation. (**C**) The level of nuclear SQSTM1 was analyzed in AT cells exposed to irradiation (IR) for 0 and 5 h in the absence or presence of leptomycin B (Lepto B) as indicated. Quantification of the mean number of nuclear SQSTM1 puncta shown in (**C**) and representative images are shown in (**D**). Scale bar: 10 µm. (**E**) Representative blot of ATR in MRC5 fibroblasts treated with siRNA as indicated. The level of nuclear SQSTM1 was analyzed in MRC5 fibroblasts treated with siRNA as indicated and exposed to irradiation (IR) for 0 and 5 h in the absence or presence of leptomycin B. Quantification of the mean number of nuclear SQSTM1 puncta shown in (**F**) and representative images are shown in (**G**). (**H**) Representative images of hepatocytes and enterocytes from male C57BL/6 wild-type mice. Sections were immunostained with antibodies against SQSTM1 and γH2AFX. Arrowheads in the zoomed merge indicate points of colocalization. Scale bar: 10 µm. (**I**) Representative images of hepatocytes from 3-, 15- and 24-month-old male C57BL/6 wild-type mice maintained under *ad libitum* (AL) or a dietary restricted (DR) diet. Sections were immunostained with an antibody against SQSTM1. SQSTM1-positive nuclei are indicated in white (**I**) and quantified in (**J**). A higher magnification of SQSTM1-positive nuclei is shown with arrowheads pointing to SQSTM1 foci. Scale bars: 10 µm.

**Figure S2.** SQSTM1 suppresses resolution of TP53BP1-positive DDF. (**A**) Representative images of TP53BP1 foci in *sqstm1^-/^*^-^ and *Sqstm1^+/+^* MEFs 0-480 min following irradiation. (**B**) Representative images showing TP53BP1 foci in *sqstm1^-/-^* and *Sqstm1^+/+^* MEFs following the induction of DNA damage with etoposide for 120 min either followed with or without a 300-min recovery period (in the absence of etoposide). (**C**) Immunoblot analyses showing the levels of SQSTM1 in *Sqstm1^+/+^*, *sqstm1^-/-^* and *sqstm1^-/-^*+FLAG SQSTM1 cell lines. Note that transgenic FLAG-SQSTM1 is expressed at lower levels than the endogenous protein. (**D**) Representative images of neutral comet analysis of *Sqstm1^+/+^*, *sqstm1^-/-^* and *sqstm1^-/-^*+FLAG-SQSTM1 following the induction of DNA damage with etoposide for 120 min either followed with or without a 300-min recovery period (in the absence of etoposide). (**E**) Representative blot of SQSTM1 in MRC5 human fibroblasts transduced with shRNA as indicated. EdU incorporation was analyzed in MRC5 human fibroblasts treated with shRNA as indicated 3, 8 and 24 h following 1 Gy X-ray irradiation quantification shown in (**F**) representative images shown in (**G**). (**H**) Representative images of TP53BP1 foci in *sqstm1^-/-^* MEFs overexpressing the indicated GFP-tagged SQSTM1 mutants non-IR and 300 min following 1 Gy X-ray irradiation. Scale bars: 10 µm; n=3.

**Figure S3.** SQSTM1 mediates the effect of autophagy on DNA repair. (**A-B**) M5-7 MEFs were treated with tetracycline (Tet) to induce knockout of *Atg5*. Representative images of GFP-TP53BP1 foci 0-480 min post-irradiation are shown in (**A**) and mean number of foci was quantified (**B**). (**C-D**) DNA damage was induced in *Atg5^+/+^* and *atg5^-/-^* MEFs by 120-min incubation with etoposide and where indicated, followed by a 300-min recovery period. The mean number of TP53BP1 foci was quantified (**C**) and representative images are shown in (**D**). (**E**) *Atg5^+/+^* and *atg5^-/-^* MEFs were treated with *Sqstm1* siRNA for 96 h. Cells were collected 5 and 300 min post-irradiation and immunostained with an antibody against TP53BP1. (**F**) Representative images of *sqstm1^-/-^* and *Sqstm1^+/+^* MEFs treated with bafilomycin A_1_ in control and irradiated cells 5 and 300 min post-irradiation. Cells were immunostained with an antibody against TP53BP1. (**G-H**) Cells were treated as for (**F**) prior to collection of cell lysates and immunoblotted for LC3 (**G**) and SQSTM1 (**H**), and for GAPDH as a loading control. Scale bars: 10 µm; n=3; error bars represent S.E.M; *, p<0.05.

**Figure S4.** SQSTM1-dependent proteasomal degradation of FLNA and RAD51. (**A**) HeLa cells were transfected with FLAG-SQSTM1 overnight and treated as indicated; either non-irradiated or irradiated, in the presence or absence of leptomycin B (Lepto B). Cells were subjected to immunoprecipitation with anti-FLAG antibody. The band indicated by a red box was identified as being present in association with SQSTM1 following irradiation. This band was identified by mass spectrometry as FLNA. (**B-C**) Quantification of immunoblot analysis of nuclear FLNA (**B**), RAD51 (**C**) normalized to LMNB1 0-480 min post-irradiation. *Atg5^+/+^* and *atg5^-/-^* MEFs were pre-incubated with MG132 where indicated for 3 h. Cells were irradiated with 1 Gy X-ray irradiation and incubated in the presence of MG132 for a further 60 min; representative blot of nuclear fractions for FLNA, RAD51, SQSTM1 shown in (**D**); LMNB1 was used as a loading control. (**E-H**) The colocalization of SQSTM1 and RAD51 was analyzed in human fibroblasts (MRC5) exposed to irradiation (IR) for 0 and 5 h in the absence or presence of leptomycin B as indicated. The mean number of nuclear SQSTM1 puncta shown in (**E**), RAD51 (**F**) and SQSTM1-RAD51 colocalization (**G**). Representative images are shown in (**H**). Scale bars: 10 µm; n=3; error bars represent S.E.M.

**Figure S5.** SQTSM1-dependent inhibition of homologous recombination. Representative images (**A**) and quantification (**B**) of RAD51 foci in *sqstm1^-/-^* and *sqstm1^-/-^*+FLAG-SQSTM1 MEFs following a 120-min incubation with etoposide and, where indicated, followed by a 300-min recovery period. (**C**) Representative images of RAD51 foci 0-480 h post-irradiation in *sqstm1^-/-^* and *sqstm1^-/-^*+FLAG-SQSTM1 MEFs treated with control or *Flna* siRNA for 96 h. (**D**) Representative images of TP53BP1 foci 0 and 300 min post-irradiation in *sqstm1^-/-^* and *sqstm1^-/-^*+FLAG-SQSTM1 MEFs treated with control or *Flna* siRNA for 96 h. (**E**) qPCR showing *SQSTM1* knockdown efficiency 48 h following siRNA treatment in human skin fibroblasts with integrated HR and NHEJ reporter as indicated, *HPRT1* (*hypoxanthine phosphoribosyltransferase 1*) was used as a loading control. n=3; error bars represent S.E.M.
