# Supplementary figures and images for: SQSTM1/p62 mediates crosstalk between autophagy and the UPS in DNA repair
Source: Autophagy. 2016 Jul 8;12(10):1917–30. doi: 10.1080/15548627.2016.1210368 (PMC5391493; doi:10.1080/15548627.2016.1210368)

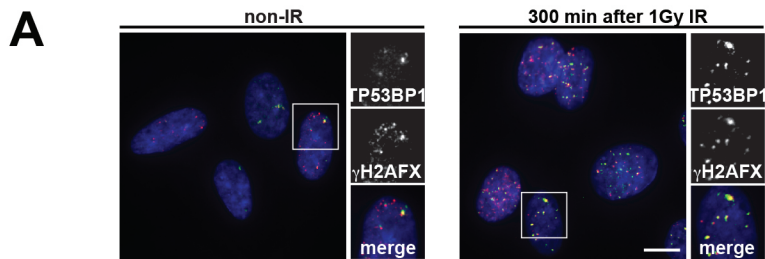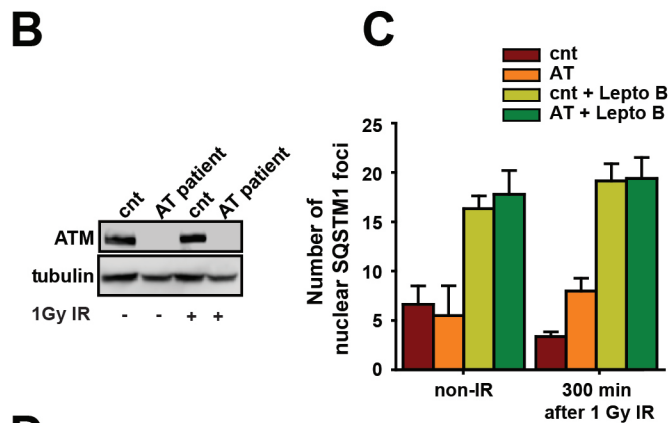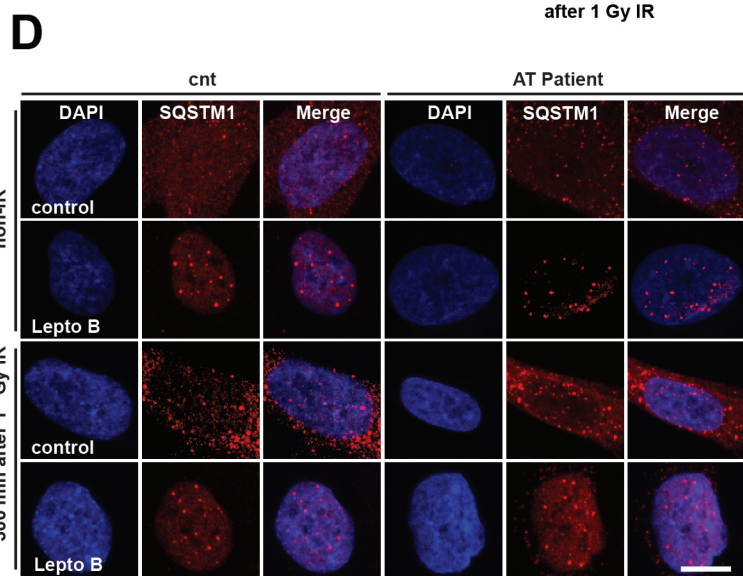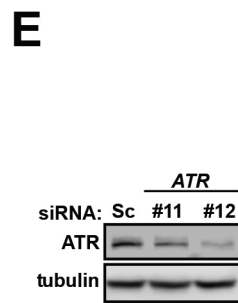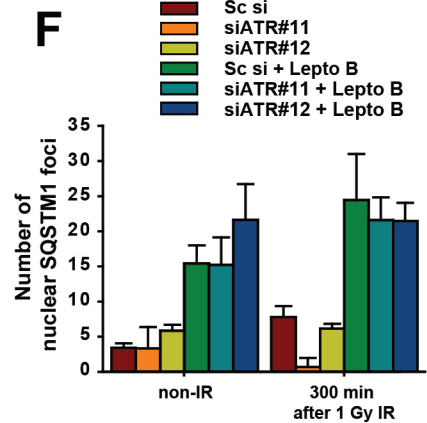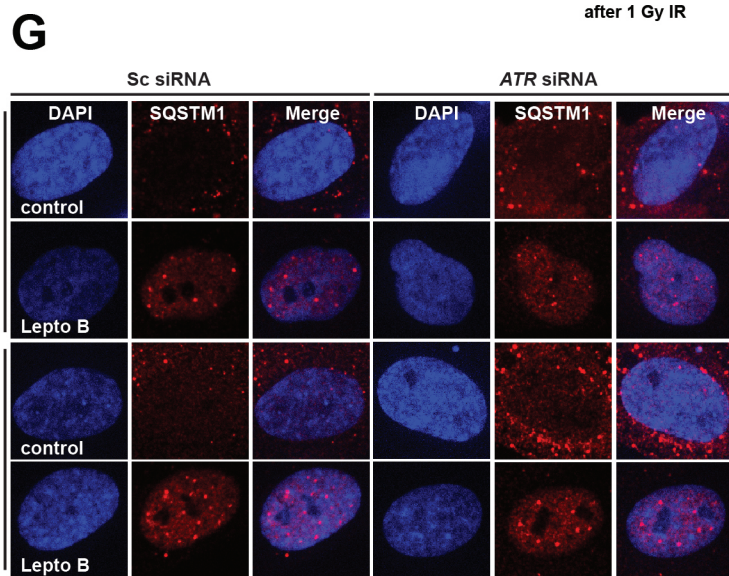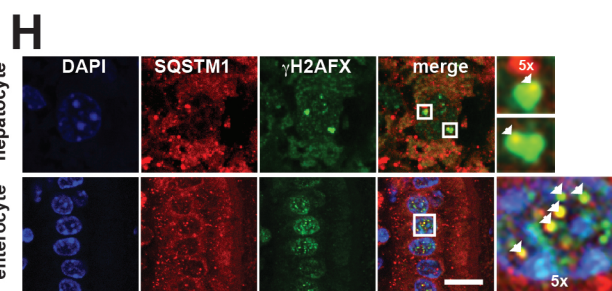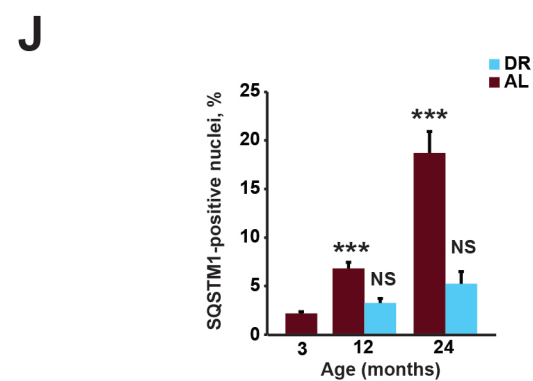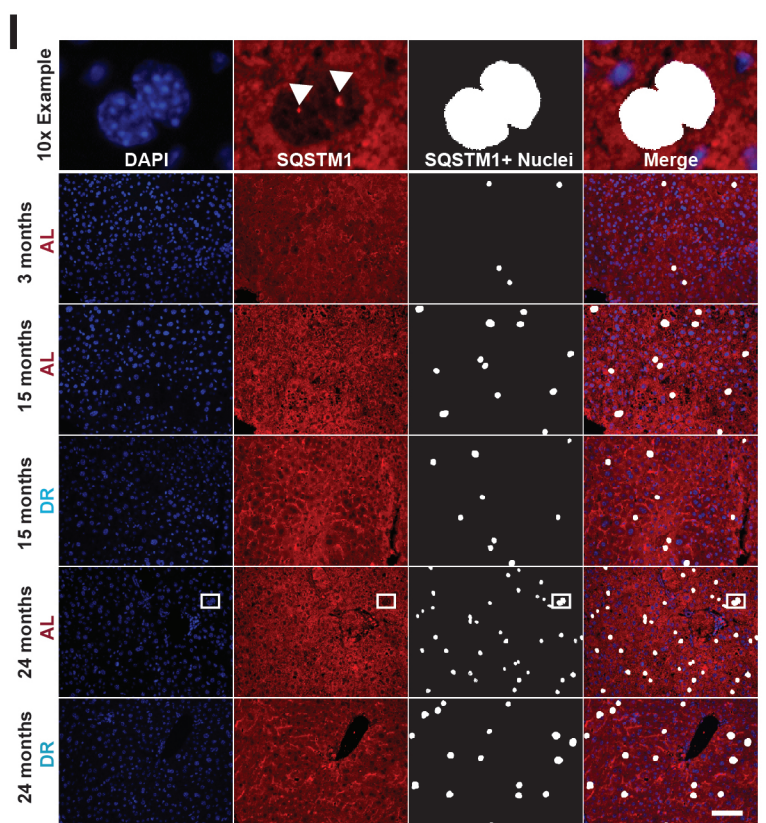

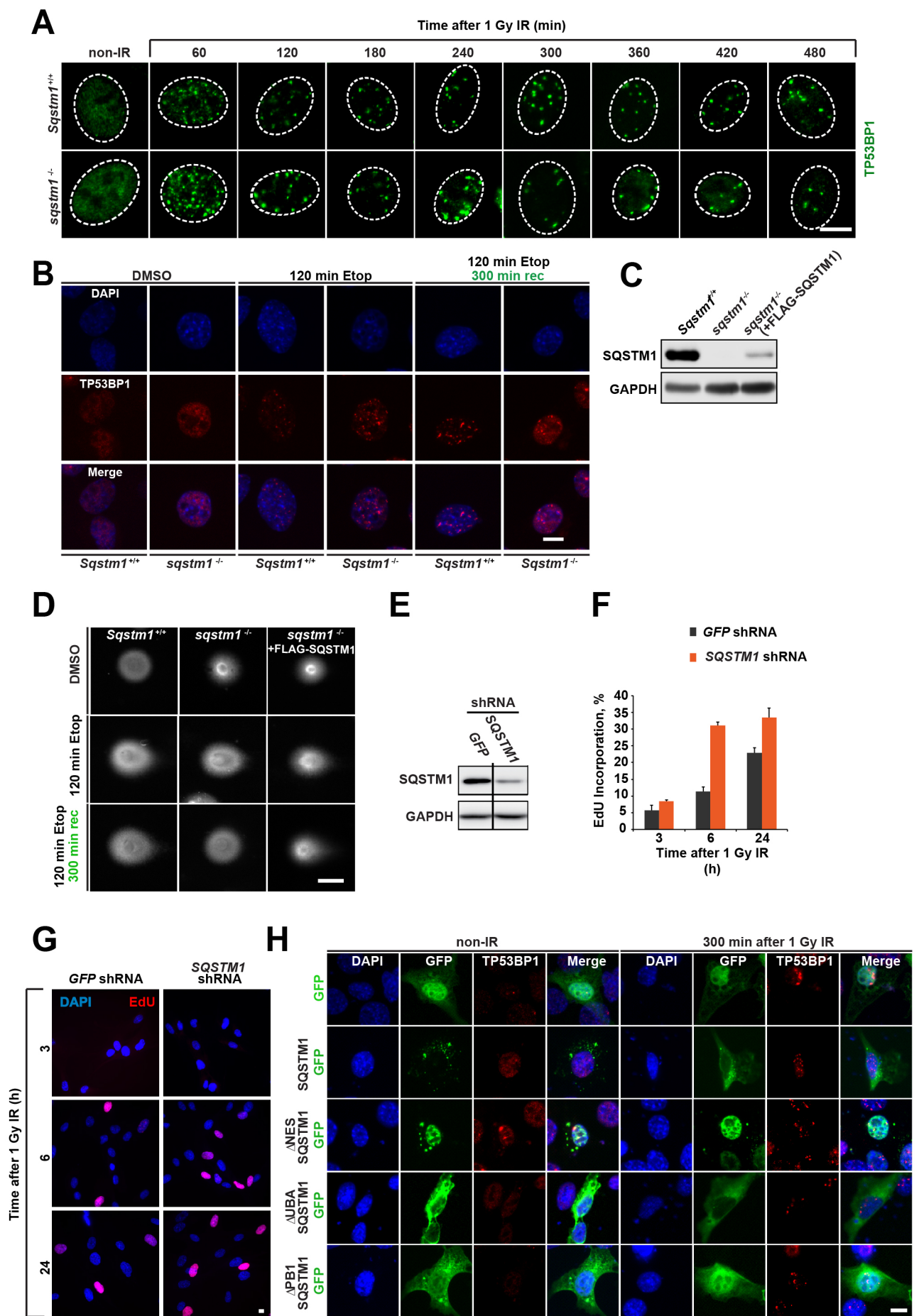

**A**

Time after 1 Gy IR (min)

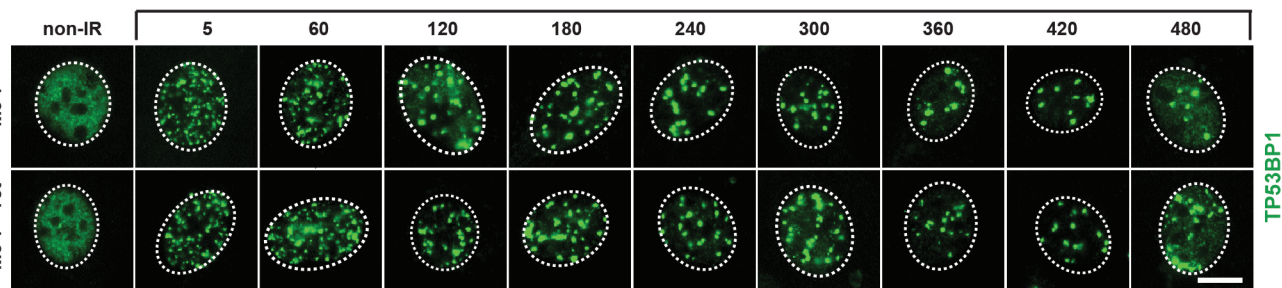**B**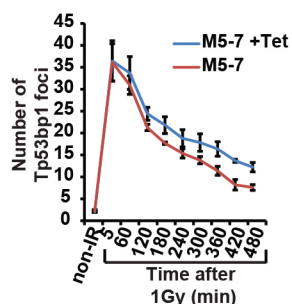**C**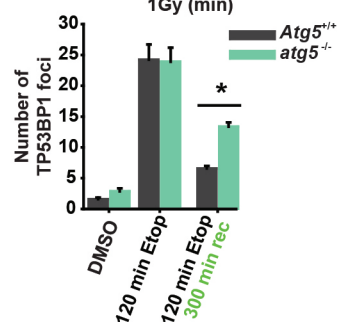**D**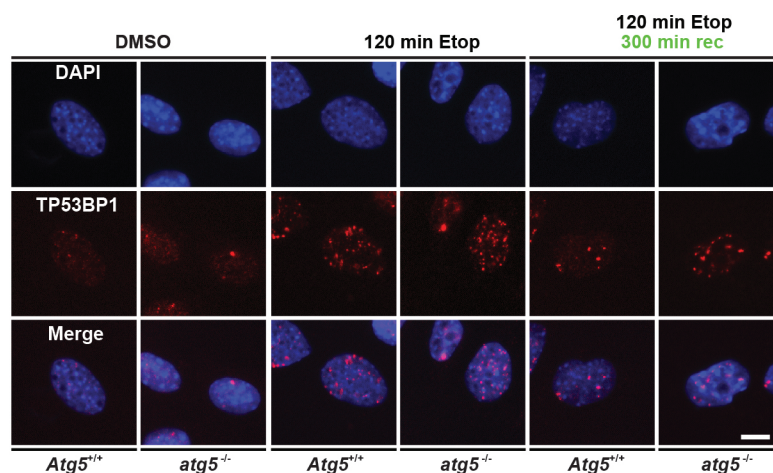**E**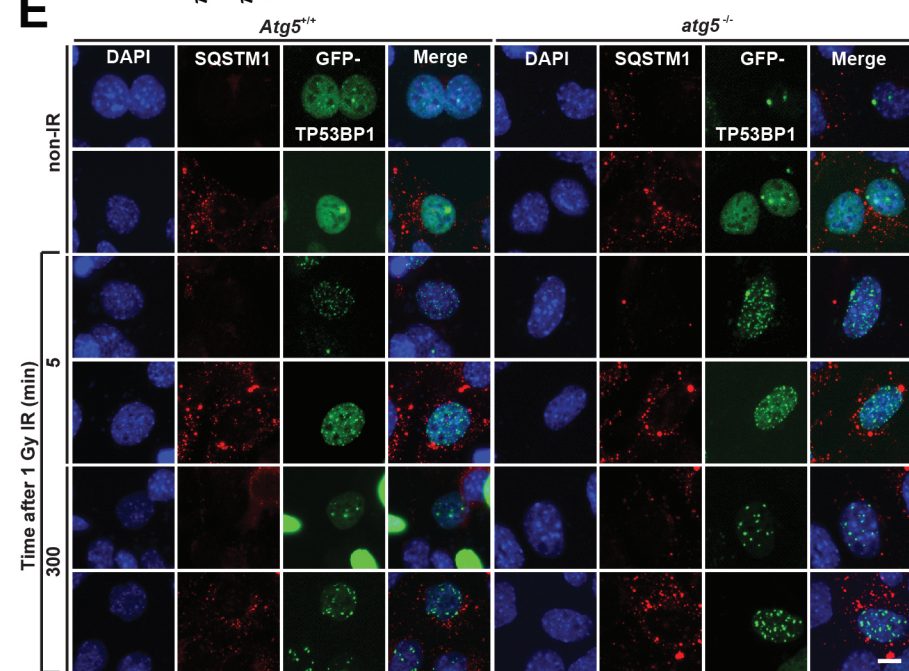**G**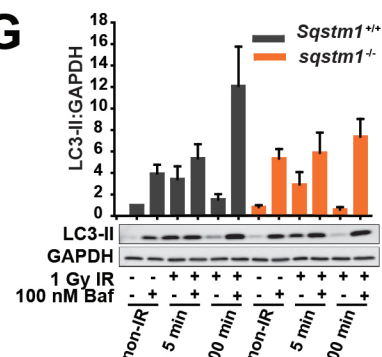**H**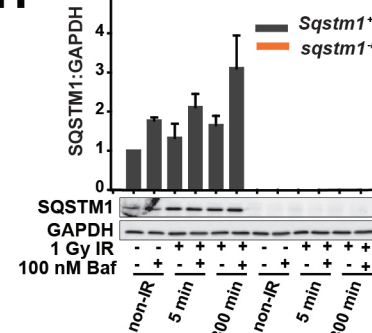**F**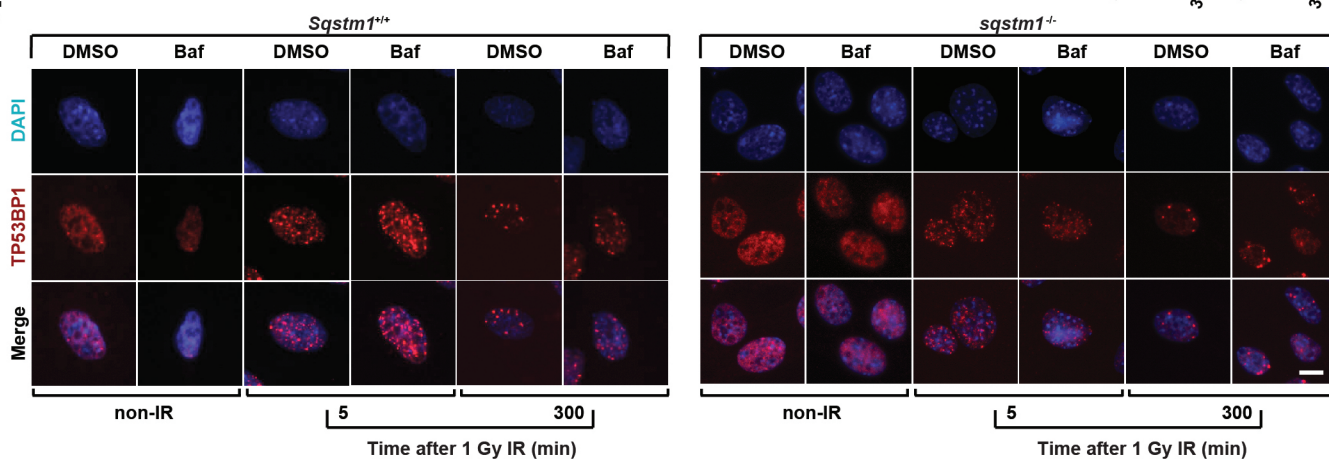

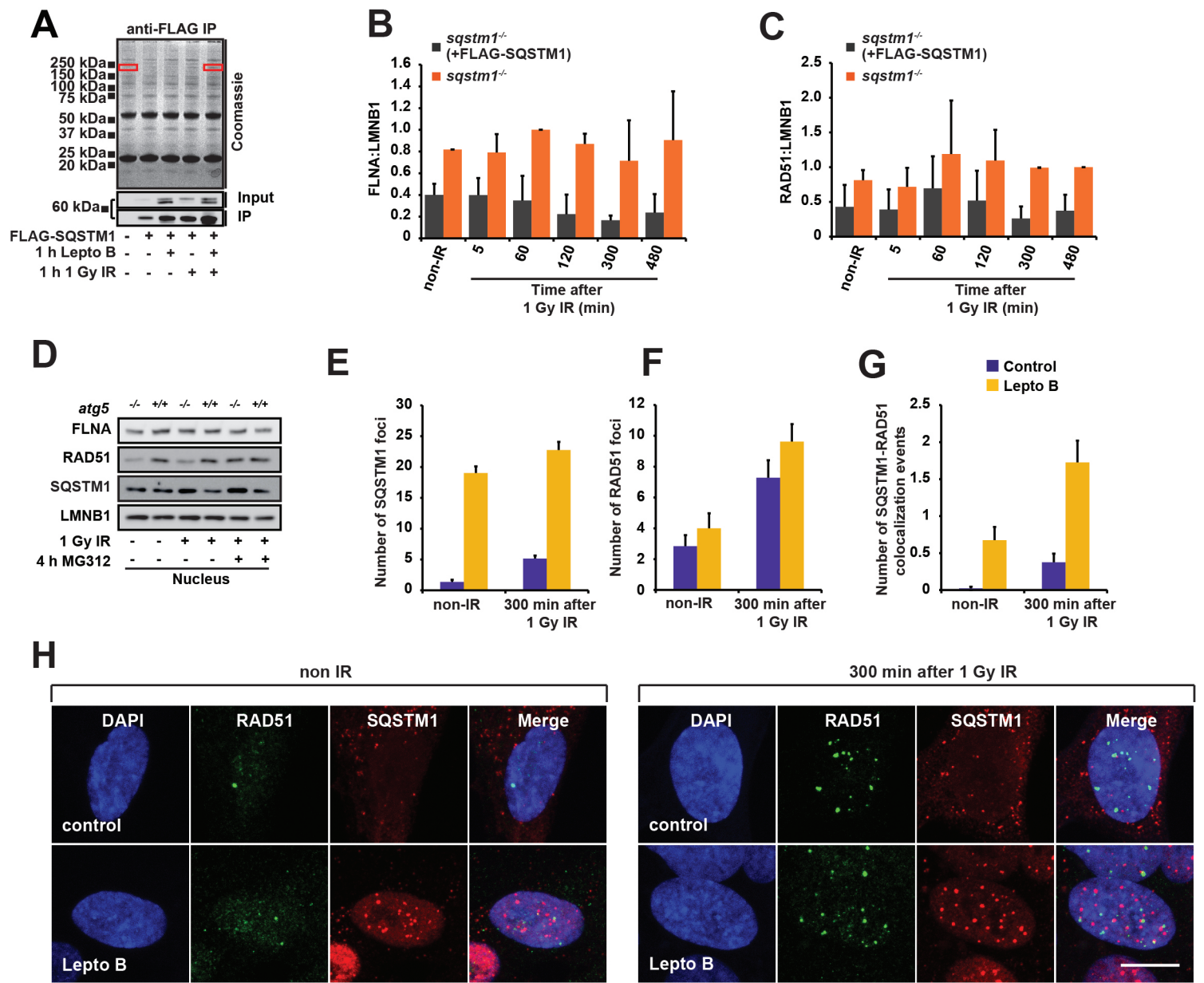

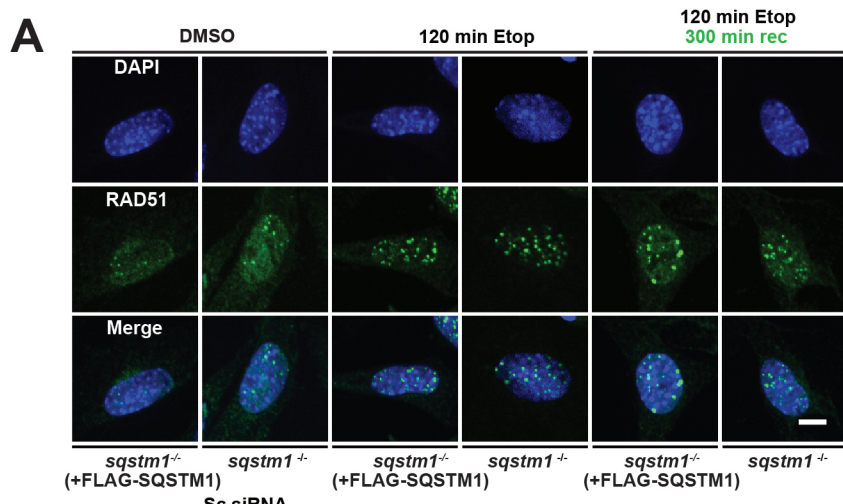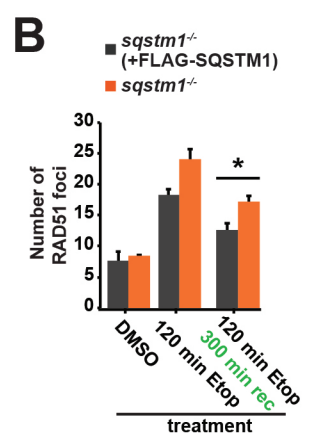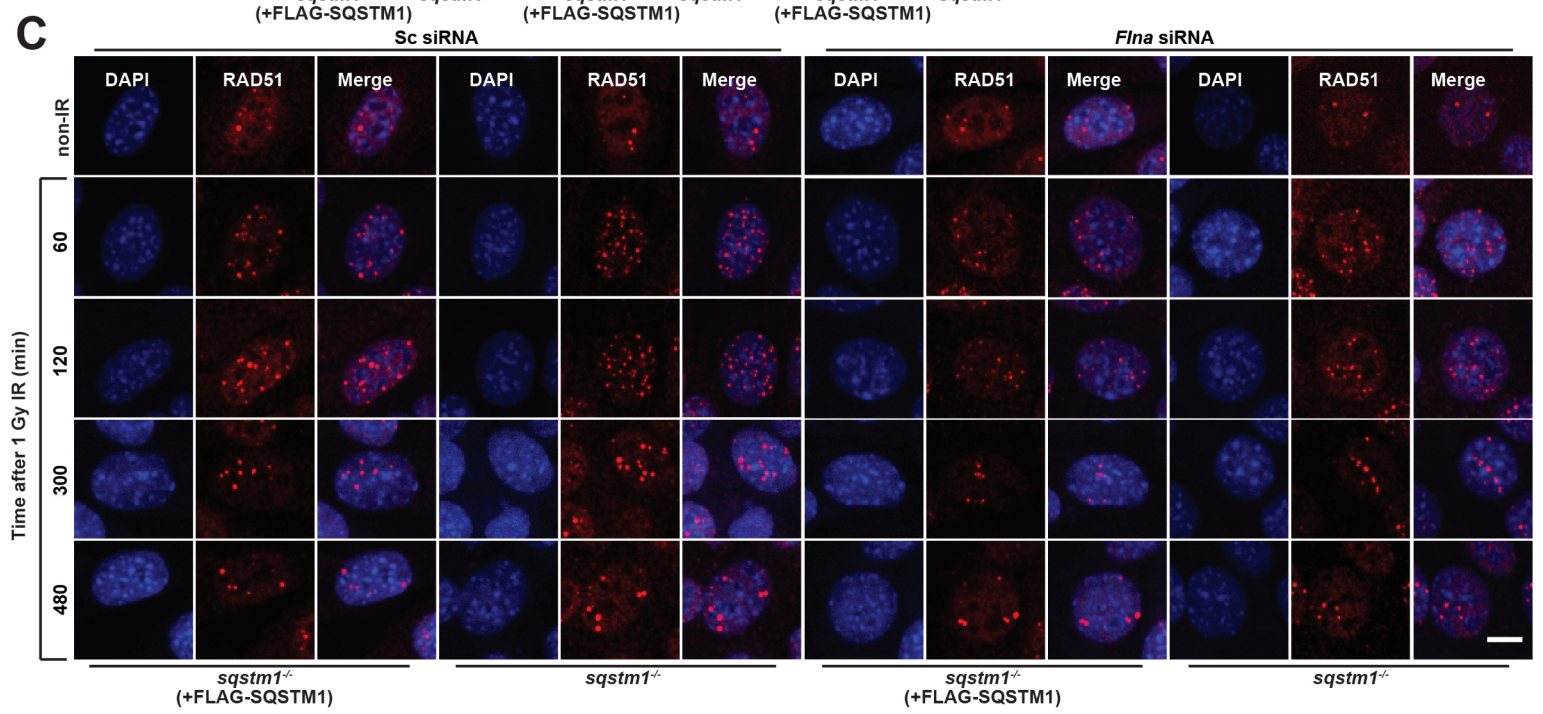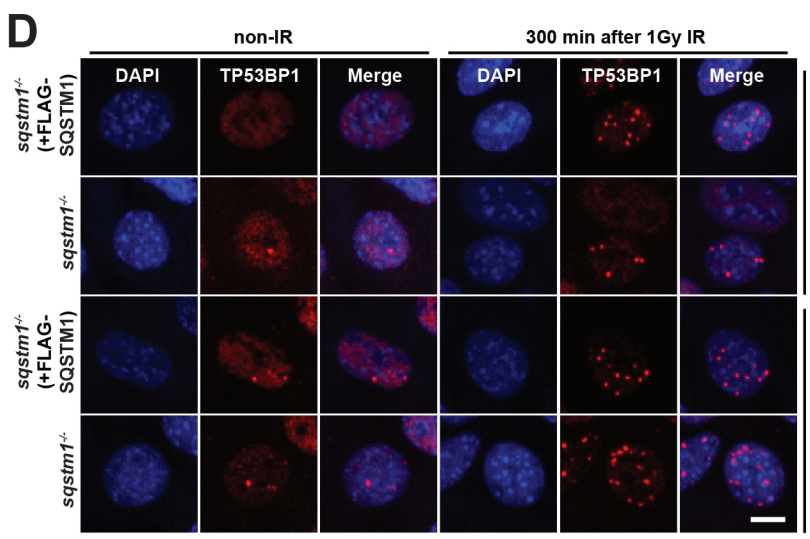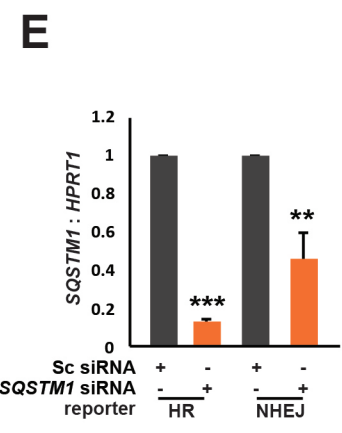

Supplement: 1210368_Supplemental_Material.zip [file kaup-12-10-1210368-s001.zip › Supplemental Figures.pdf]
